# Supplementary material for: Automatic landmarking identifies new loci associated with face morphology and implicates Neanderthal introgression in human nasal shape
Source: Commun Biol. 2023 May 8;6:481. doi: 10.1038/s42003-023-04838-7 (PMC10167347; doi:10.1038/s42003-023-04838-7)
Supplement: Supplementary file 2 — Supplementary Material [file 42003_2023_4838_MOESM2_ESM.pdf]

Automatic landmarking identifies new loci associated with face morphology and implicates Neanderthal introgression in human nasal shape

Table of contents

Supplementary Note 1: Regional association plots for genomic regions replicated in this study. 3

Supplementary Note 2: Regional association plots for novel regions detected in this study which reached genome-wide significance and replicated in either Chinese or European cohort, besides the 5 highlighted ones in the main text. 10

Supplementary Note 3: Regional association plots for novel regions detected in this study whose replication P-values are insignificant in both Chinese and European cohorts. 21

Supplementary Figures 24

- Supplementary Figure 1: Full Face++ landmarking protocol. 24
- Supplementary Figure 2: Histograms for 5 Inter-landmark distances that are most significantly associated with 5 the novel genomic regions in Table 2. 25
- Supplementary Figure 3: Covariates correlations and heritability. 26
- Supplementary Figure 4: Comparison of the results of this current study with three previous analyses of the same cohort. 27
- Supplementary Figure 5: Illustration of inter-landmark distances associated with the 6 most robustly replicated signals (*EDAR*, *GLI3*, *PAX3*, *FOXD1*, *RUNX2*, *DCHS2*). 28
- Supplementary Figure 6: Continental ancestry proportions in archaic introgression near *ATF3*. 29
- Supplementary Figure 7: GO enrichment analysis and comparison of expression levels for transcripts nearest to the novel index SNPs. 30
- Supplementary Figure 8: Boxplot of lambda values for GWASs of 148 facial distances based on nine different analysis models, using PLINK, GCTA, GENESIS, TRACTOR, or SNP1. 31
- Supplementary Figure 9: Comparison of SNP -log(P-values) obtained by PLINK with values obtained using GCTA GENESIS, TRACTOR, and SNP1 (across 148 associated distances). 32
- Supplementary Figure 10: Violin plots of -log(P-values) obtained using PLINK, GCTA, GENESIS, TRACTOR, and SNP1 for index SNPs at six well-established face gene regions (Table 1) across 148 facial distances. 33

Supplementary Tables 34

- Supplementary Table 1: Interclass correlation coefficient and median distance for each corresponding landmark pair from Face++, Dlib and manual protocols. 34
- Supplementary Table 2: Description of the 34 well-defined Face++ landmarks grouped by regions that used in the inter-landmarks distances calculation. 34
- Supplementary Table 3: Covariates correlations and narrow-sense heritabilities. 34
- Supplementary Table 4: Features of the regions showing genome-wide significant association in this study. 34
- Supplementary Table 5: Follow-up of the 33 novel face loci in the mouse. 34

Supplementary Table 6: Features of tested admixture mapping segments in this study. 34
Supplementary Table 7: Nasal height (NLH) in Neanderthal and three modern human populations. 34
Supplementary Table 8: Summary of GWAS associated regions between FDR threshold (1.82×10^{-6}) and nominal GWAS threshold (5×10^{-8}). 34
Supplementary Table 9: Summary of condition analysis to determine if the signal is reported previously. 34
Supplementary Table 10: Summary of genomic inflation factor (lambda) from various GWAS models. 34
Supplementary Table 11: Cell type classification of RNA-seq samples from ENCODE used in study. 34
Supplementary Movie: Mouse craniofacial morphology impacted by 22q12.1 (index SNP: rs32069343). 34

Supplementary Note 1: Regional association plots for genomic regions replicated in this study.

Some of those regions are associated with multiple inter-landmark distances (ILDs) (see Supplementary Table 4). Below we only show plots for the trait with the strongest association to each region.

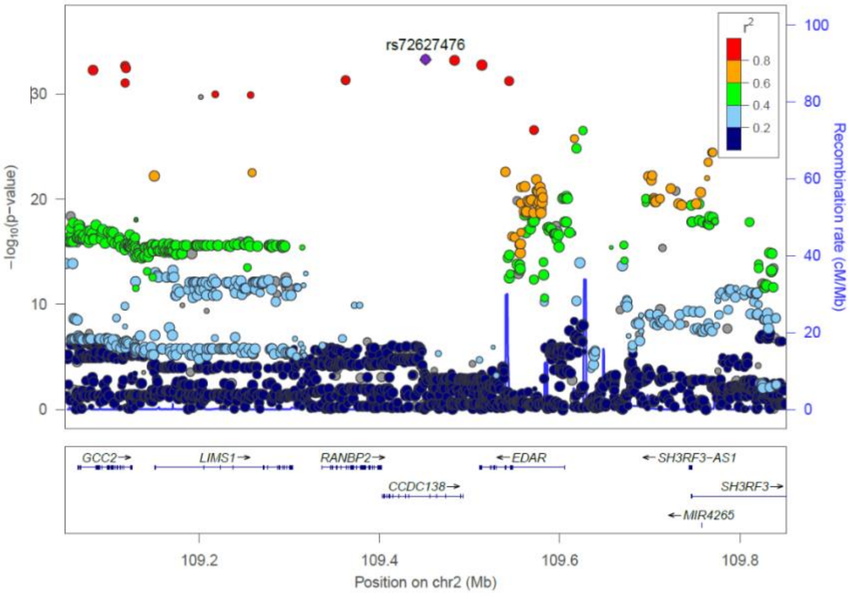

2q12.3: *EDAR*. The most robustly replicated region involves SNPs in strong LD over a segment of >10Mb in 2q12.3 centered around the *EDAR* (Ectodysplasin A Receptor) gene. SNPs in this region have been previously associated with facial morphology in Latin Americans and East Asians, particularly in relation to facial flatness, jaw protrusion, and distance between ectocanthion and otobasion inferius[1-3]. Overall, we observe that 1,245 SNPs in 2q12.3 are associated with 75 ILDs. The strongest association was detected for D198 (involving landmarks 9-23, P-value 5.6×10^{-34}, Supplementary Figure 5). The associated SNPs include rs3827760, which encodes a functional amino acid substitution in *EDAR*, the derived allele being absent in Europeans, but having a high frequency in East Asians and being fixed in Native Americans (resulting in a frequency in the CANDELA sample of 0.41). Most of the distances showing association with the *EDAR* region are strongly correlated, and involve landmarks around the eye and eyebrow (Supplementary Figure 5). A previous facial hair genome-wide association study (GWAS) in CANDELA showed a suggestive association between *EDAR* and eyebrow thickness[4], thus some of the associations seen here could be influenced by variation in eyebrow size. In addition, some associated distances involve landmarks around the nose or mouth, consistent with a previous study in Eurasians[3]. Overall, the derived allele (G) of rs3827760 is associated with an increase of eyebrow and eye size, and a smaller distance between the eyes.

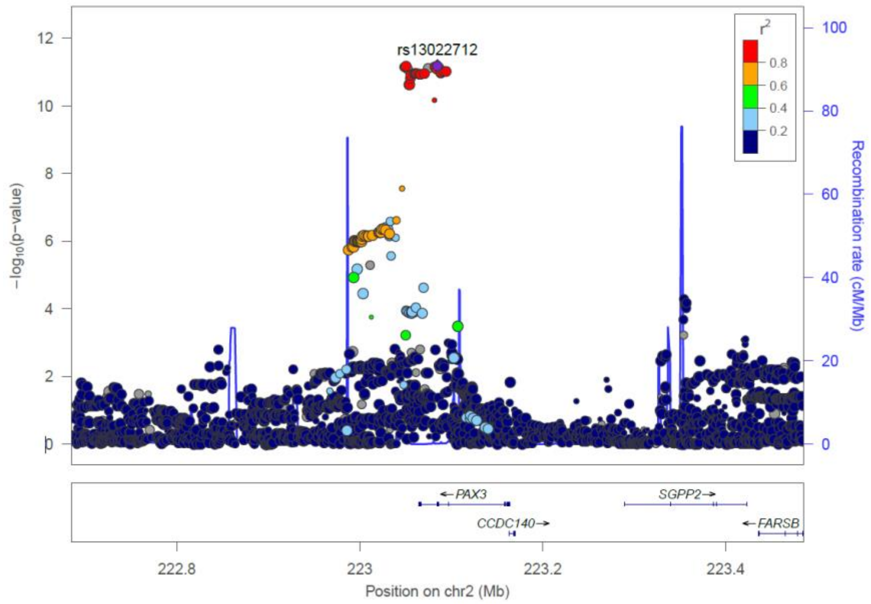

2q36.1: *PAX3*. *PAX3* (Paired box 3) (2q36.1, P=2.04×10^{-10}), is the most frequently replicated locus across studies of facial morphology, so far having been associated in eight independent GWAS[2, 3, 5-10], always in relation to measures sensitive to nasion position. We previously detected a *PAX3* association in GWAS of the CANDELA 2D photographs, using both categorical phenotyping and measures derived from manual landmarking[1, 2]. In addition, SNPs in *PAX3* have been associated with the distance between eyes, and with brow ridge protrusion[2, 6]. Here we observe association of SNPs in this region with 18 distances, mostly involving the nasion and eyebrow area. In addition, some associated distances are sensitive to nose height (Supplementary Figure 5). The 18 distances associated with *PAX3* are strongly correlated. The strongest association is seen for rs13022712 with D99 (landmarks 9-16), and this index SNP is located within an intron of *PAX3*. The minor allele of the index SNP in this region (rs13022712) is associated with a shortening of the nose/mid-face. *PAX3* has been shown to play a key role in fetal development, particularly in relation to neural development and myogenesis[11].

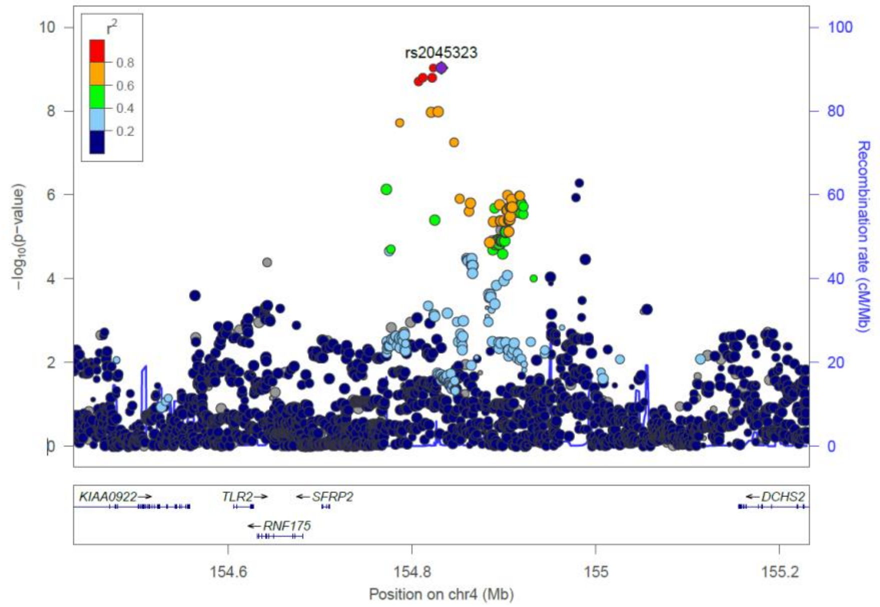

4q31.3: Intergenic (*DCHS2, SFRP2*). SNPs in 4q31.3 in the vicinity (76 Kb away) of the *SFRP2* (secreted frizzled related protein 2) and (234 Kb away) *DCHS2* (dachsous cadherin-related 2) genes, have been reported to be associated with endocanthion-alare distance[10], columella inclination[1] and nose morphology[5]. Here we find association of this region with 8 distances, including endocanthion-alare (D205: landmarks 16-23). We also find a strong association of SNPs in this region with nose morphology. Furthermore, two previously reported index SNPs (rs6535972 and rs9995821), show genome-wide significant association with at least one distance obtained here with Face++ landmarks. The minor (A) allele of the SNP (rs2045323) with the smallest P-value obtained here (P-value 9.35×10^{-10}), is associated with a narrower and longer nose (mainly resulting from a lower position of the alare landmark, Supplementary Figure 5). *SFRP2* has been shown to have important roles in craniofacial development in mice[12]. Furthermore, cartilage defects have been observed in zebrafish embryos deficient in *Dchs2*[13].

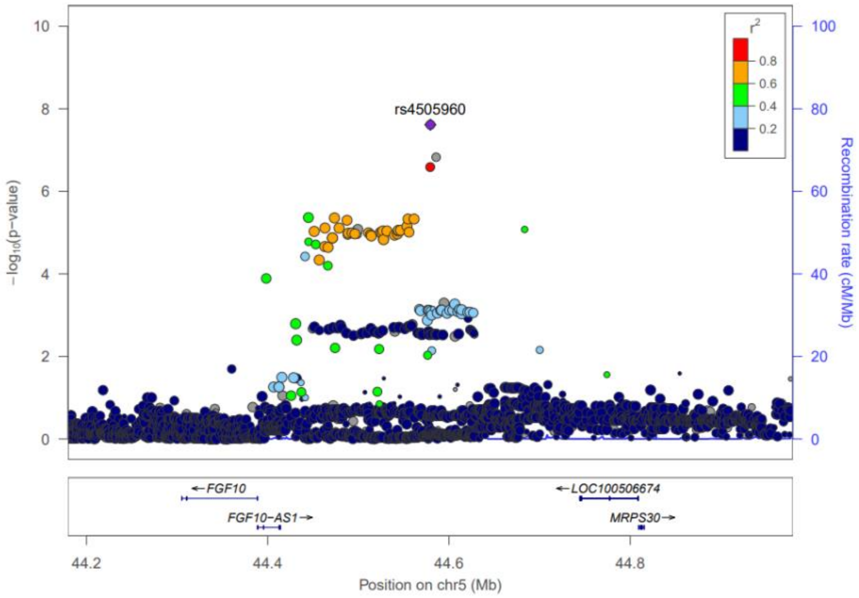

5p12: Intergenic (*FGF10, LOC100506674*). A region of 5p12 about 190 Kb away from *FGF10* (Fibroblast Growth Factor 10), has been reported to be associated with variation in a temporal facial segment, using a 3D multivariate approach[9]. Here, we detected an association of SNPs in this genomic region with three ILDs (the most significant one is the distance between right alare and left eyebrow lower right corner). Rare mutations in *FGF10* have been shown to cause Lacrimo-auriculo-dento-digital syndrome (LADDS)[14], an autosomal dominant ectodermal dysplasia associated with facial dysmorphology.

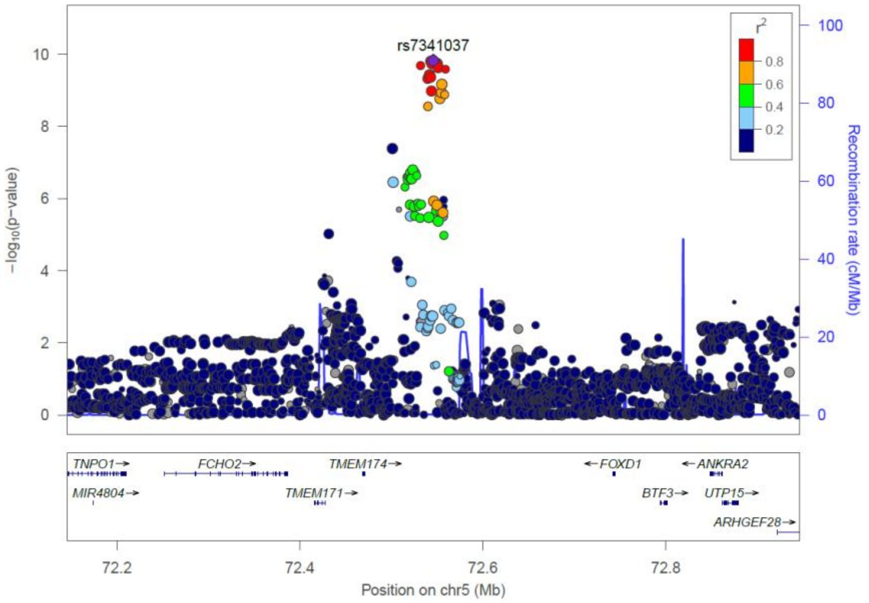

5q13.2: *FOXD1*. SNPs in 5q13.2 located 182 Kb away from *FOXD1* (forkhead box D1) show association with 10 ILDs in the eye/eyebrow area. Six of these distances are sensitive to eyebrow size (thickness and length), with the other four being sensitive to either eye size, or spacing distance between eye and eyebrows. *FOXD1* has been associated with eyebrow thickness in a GWAS in a Chinese sample[15] and we previously observed suggestive association with the same trait evaluated qualitatively in the CANDELA

sample[4, 15]. *FOXD1* has been shown to be involved in hair growth[16]. The minor allele (C) of the index SNP in this region (rs7341037) is associated with larger eyebrows.

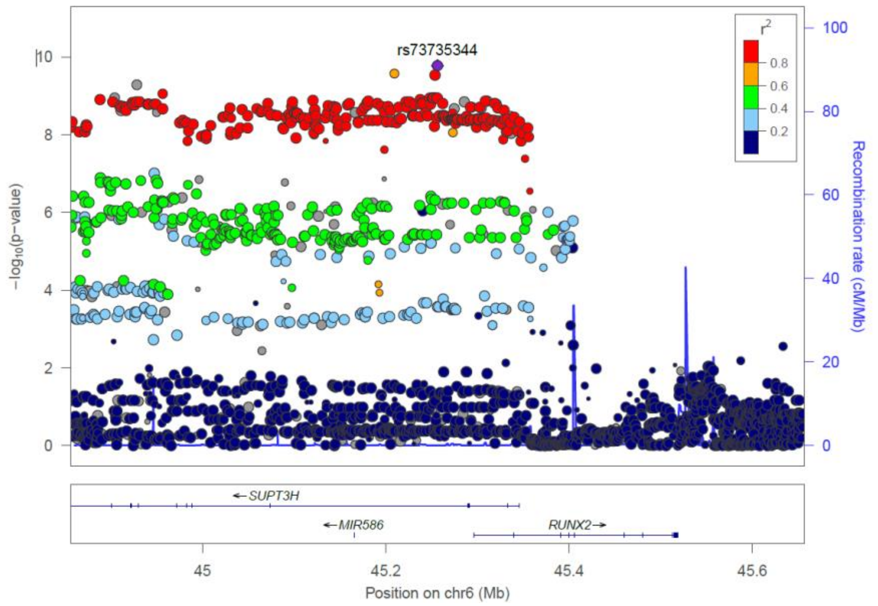

6p21.1: *SUPT3H, RUNX2*. We previously reported association of SNP in 6p21.1 overlapping *RUNX2* (RUNX Family Transcription Factor 2) and *SUPT3H* (SPT3 Homolog) with nose bridge breadth[1], forehead protrusion, brow ridge protrusion and upper face flatness[2]. Subsequently, GWAS in other study samples have reported association of SNPs in this region with nose morphology, chin dimples, and distance between right entocanthion and left otobasion inferius[3, 5, 8]. Here we observe association of *RUNX2*/*SUPT3H* with 15 distances, involving landmarks on the eyebrows and mouth, thus generally sensitive to variation in midface height. The strongest association is seen for rs141680515 with D99 (landmarks 9-16), and rs141680515 is intronic to *SUPT3H*. Previous studies have shown an evolutionary effect of *RUNX2* variation on facial length in carnivores[17]. Furthermore, *RUNX2* is known to be involved in osteoblastic differentiation and skeletal morphogenesis[18-20], and *RUNX2*/*SUPT3H* has been associated with Cleidocranial Dysplasia and familial Hepatic Adenomas[21, 22].

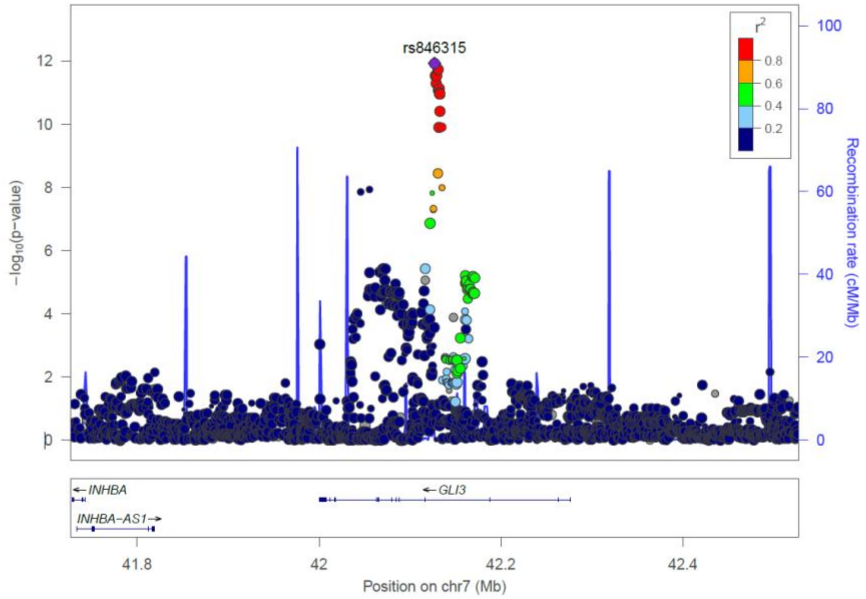

7p14.1: *GLI3*. Associated SNPs in 7p14.1 overlap the *GLI3* (GLI Family Zinc Finger 3). We previously reported an association of SNPs in this region with nose wing breadth assessed qualitatively and through manual landmarking[1]. Consistent with our previous findings, the automatic landmarking performed here revealed an association of SNPs in this region with the distance between right and left alare, and the distance between left and right nasal root contour. The minor allele of the index SNP (rs846315), an intron variant, is associated with a wider nose. *GLI3* has been shown to play a key role in embryogenesis[23], mutations in *GLI3* causing Greig cephalopolysyndactyly and Pallister-Hall syndromes[24, 25].

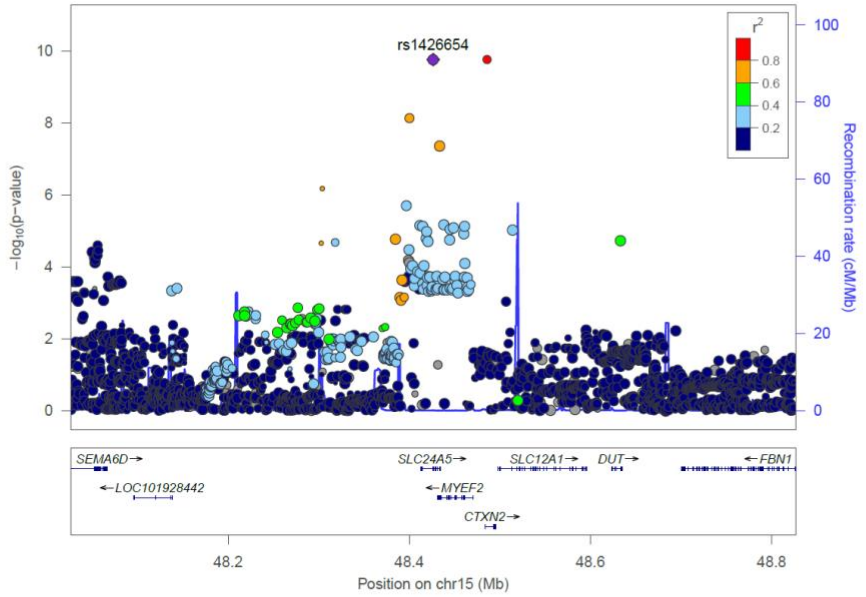

15q21.1: *SLC24A5*. Based on the manual landmarking of CANDELA profile photographs, we previously reported an association signal in 15q21.1, overlapping the *SLC24A5* (Solute Carrier Family 24 Member 5) gene, with nose tip roundness (and suggestive significantly associated with columella inclination and lower lip thickness)[2]. The automatic landmarking of the frontal CANDELA photographs performed here

revealed an association of this region with upper lip thickness (D527: landmarks 31-33). The index SNP (rs1426654) showed the strongest association in this and in our previous study. This SNP encodes a nonsynonymous amino-acid change in exon3 of *SLC24A5*. The alternative allele is absent in Europeans, but has an extremely high frequency in East Asians (0.97), Native Americans (0.98), and Africans (0.99), which results in an intermediate frequency in the CANDELA sample of 0.43. This index SNP has a positive effect on both ILDs, which would cause lip thickness increases.

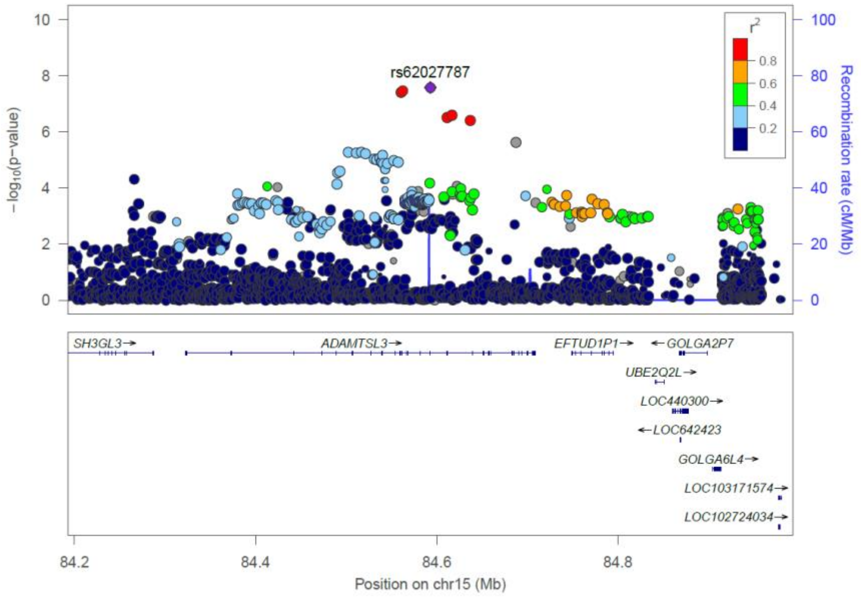

15q25.2: *ADAMTSL3*. SNPs in 15q25.2 overlapping *ADAMTSL3* (ADAMTS like 3) have been reported to be associated with variation on a GWAS of 3D of facial variation using a multivariate phenotyping approach[9]. Here we observe association of this region with distances between landmarks 8 and 23, reflecting variation between eye and eyebrow. The alternative allele of the index SNP (rs62027787) is absent in East Asians and Africans, and has a relatively low frequency in Native Americans (0.002) and relatively high frequency in Europeans (0.14), which results in a frequency in CANDELA samples of 0.076. One of the SNPs associated here (rs34047645), encodes a nonsynonymous substitution in exon18 of *ADAMTSL3*. This gene has been shown to impact on the proliferation of hepatocellular carcinoma cells[26].

Supplementary Note 2: Regional association plots for novel regions detected in this study which reached genome-wide significance and replicated in either Chinese or European cohort, besides the 5 highlighted ones in the main text.

Some of those regions are associated with multiple ILDs (see Supplementary Table 4). Below we only show plots for the trait with the strongest association to each region.

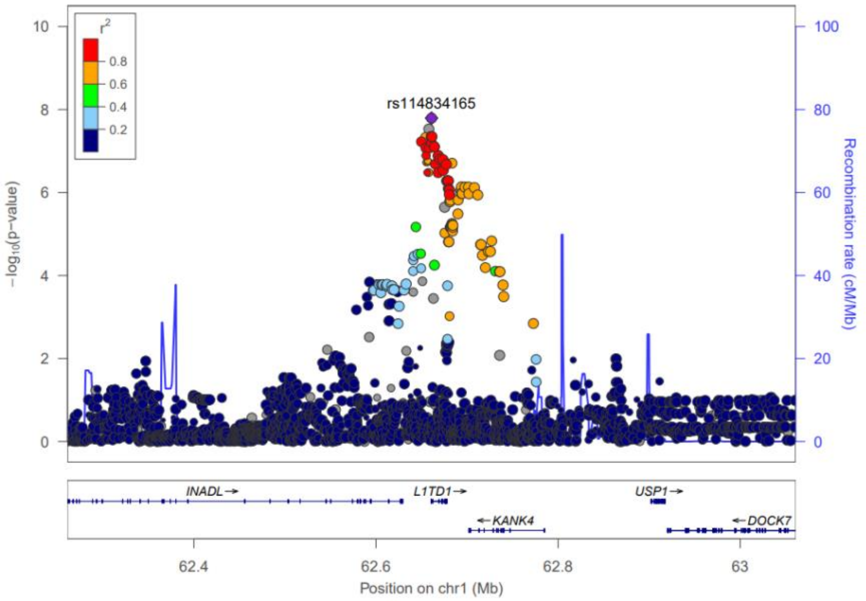

1p31.3: *KANK4*. We associated SNPs from *L1TD1* (LINE1 type transposase domain containing 1) and *KANK4* (KN motif and ankyrin repeat domains 4) region with three ILDs and the most significant one is the distance between left frontotemporale and right exocanthion. Homozygous mutations of *KANK4* gene were identified in two patients with nephrotic syndrome, and they also presented facial dysmorphism and short stature (OMIM #614612).

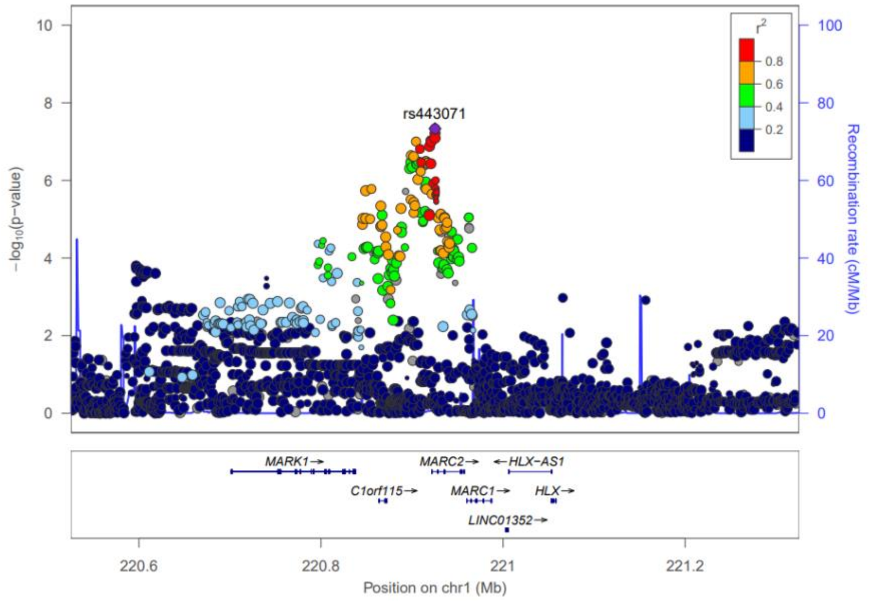

1q41: ***MARK1, MARC2.*** We associated an SNP from *MARK1* (microtubule affinity regulating kinase 1) and *MARC2* (Mitochondrial amidoxime reducing component) region with the distance between subnasal and stomion. *MARK1* has been reported to be associated with a facial segment previously[9]. However, according to the conditioning analysis, our signal is independent of the signal reported before.

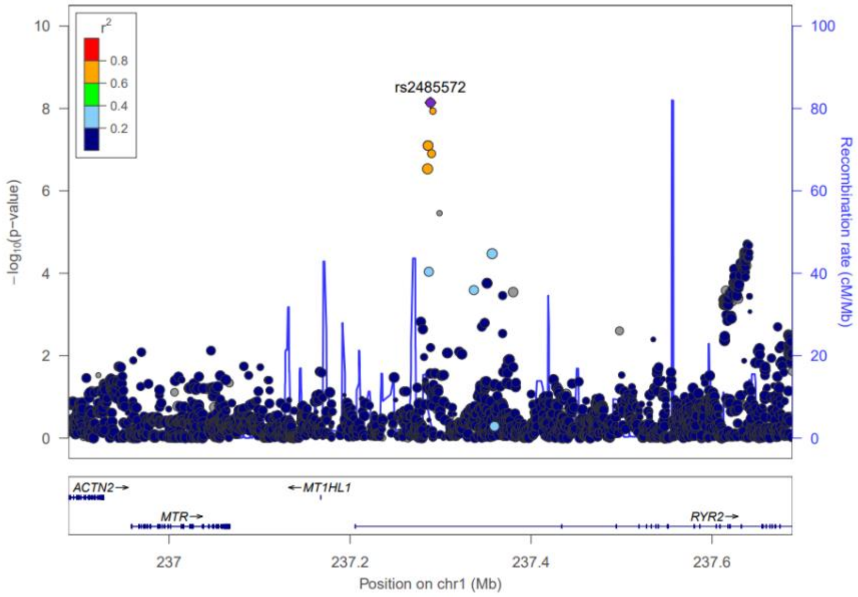

1q43: ***RYR2.*** We associated SNPs within *RYR2* (ryanodine receptor 2) gene with three ILDs and the most significant one is the distance between right exocanthion and stomion. Mutations in *RYR2* gene were reported to be associated with stress-induced polymorphic ventricular tachycardia[27].

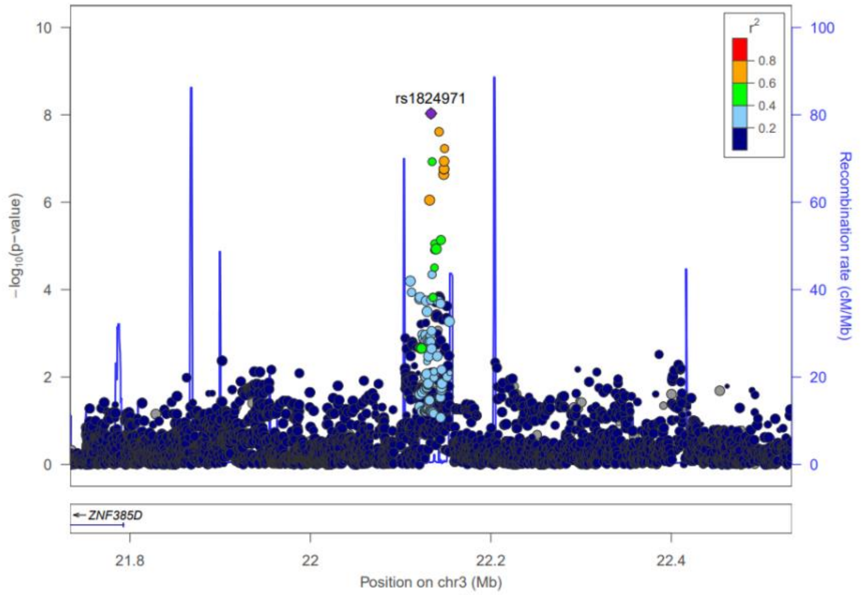

3p24.3: Intergenic (*ZNF385D*). We associated SNPs in 3p24.3 region with eyebrow thickness.

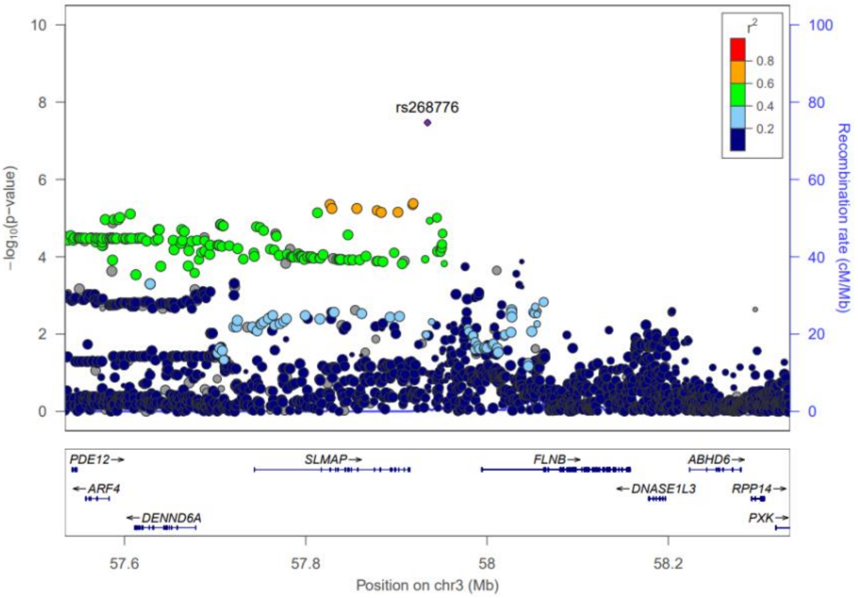

3p14.3: Intergenic (*SLMAP, FLNB*). We associated SNPs from *SLMAP* (sarcolemma associated protein) and *FLNB* (filamin B) region with eyebrow thickness. It was reported that a patient with *FLNB* gene exclusion presented postnatal growth retardation, facial dysmorphism, and other abnormal traits[28].

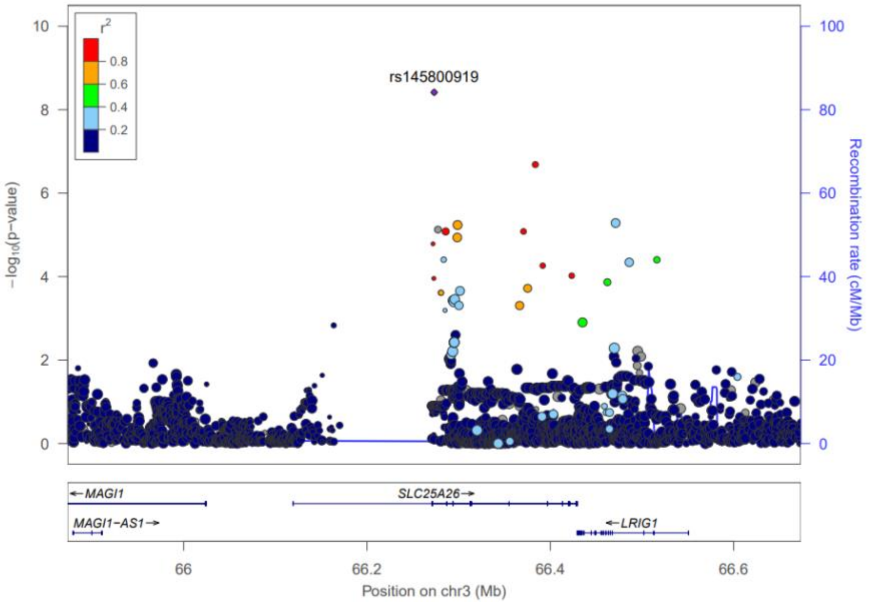

3p14.1: *SLC25A26*. We associated one SNP within *SLC25A26* (solute carrier family 25 member 26) with two ILDs and the most significant one is the distance between right cheilion and left palpebrale inferious.

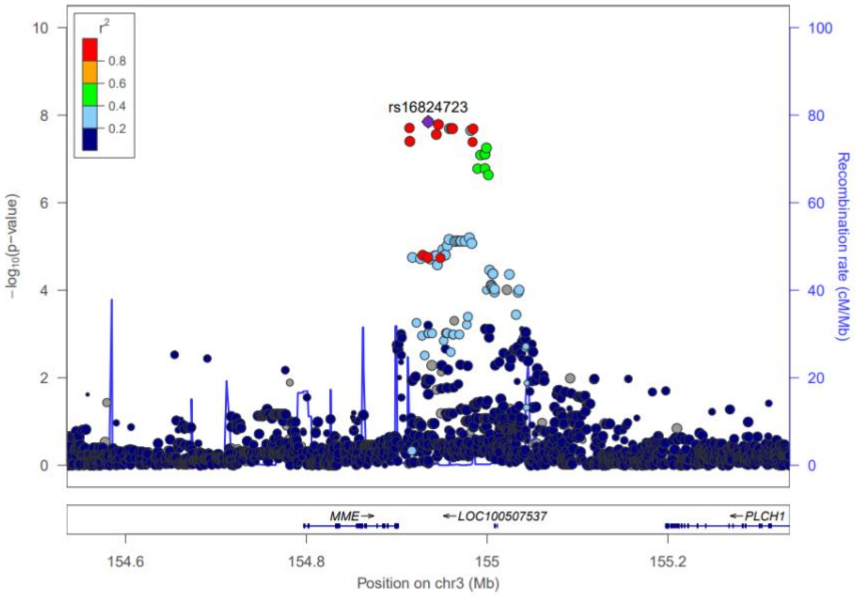

3q25.2: *MME*, *LOC100507537*. We associated SNPs from *MME* (membrane metalloendopeptidase) and *LOC100507537* with four ILDs that are all related to eyebrow size. It is reported that mutations in *MME* would cause Charcot-Marie-Tooth disease, and they showed muscle weakness, atrophy, and sensory disturbance in the lower extremities[29].

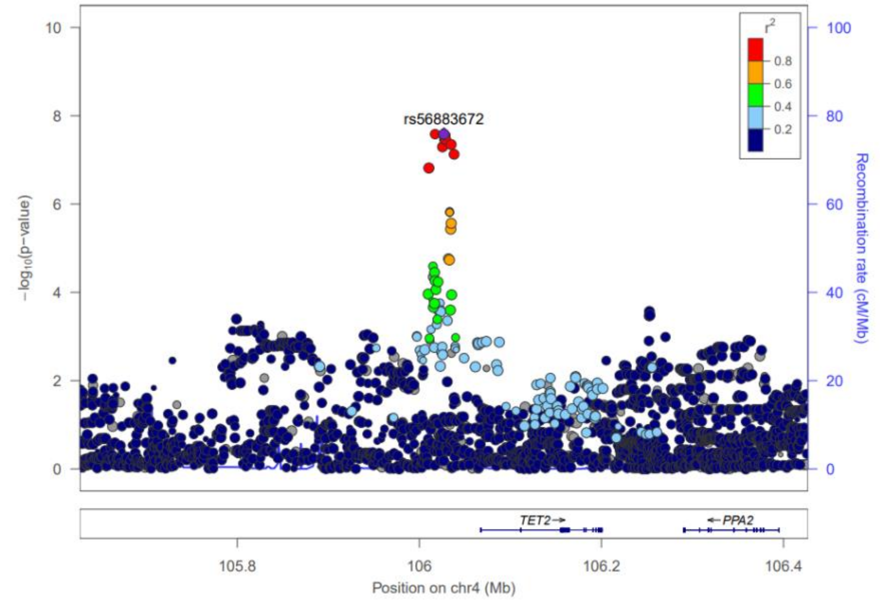

4q24: Intergenic (*TET2*). We associated one SNP in 4q24 region with the distance between the frontozygomatic suture and the uppermost landmark of the face contour.

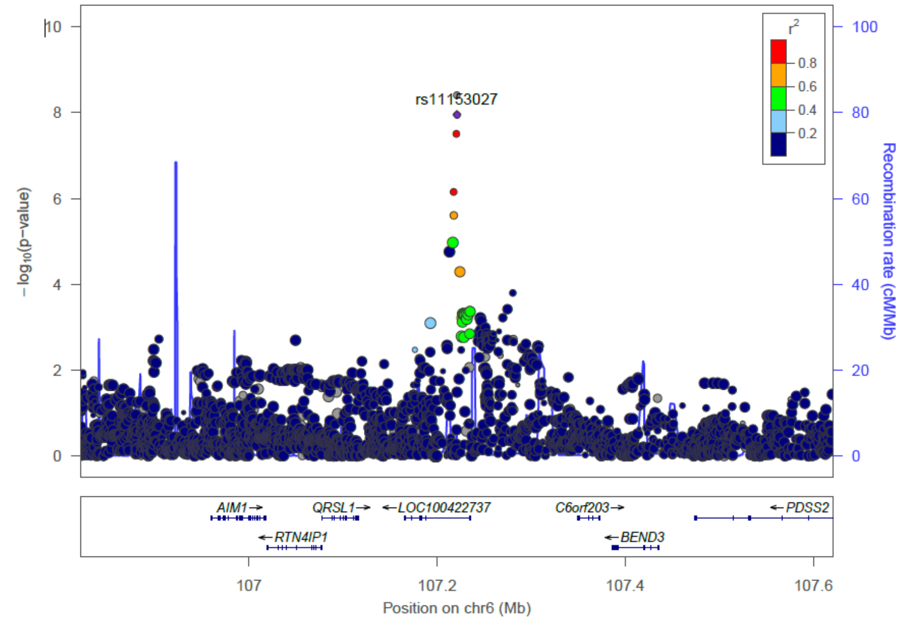

6q21: *LOC100422737*. We associated SNPs within *LOC100422737* with three ILDs and the most significant one is the distance between alare and subnasal.

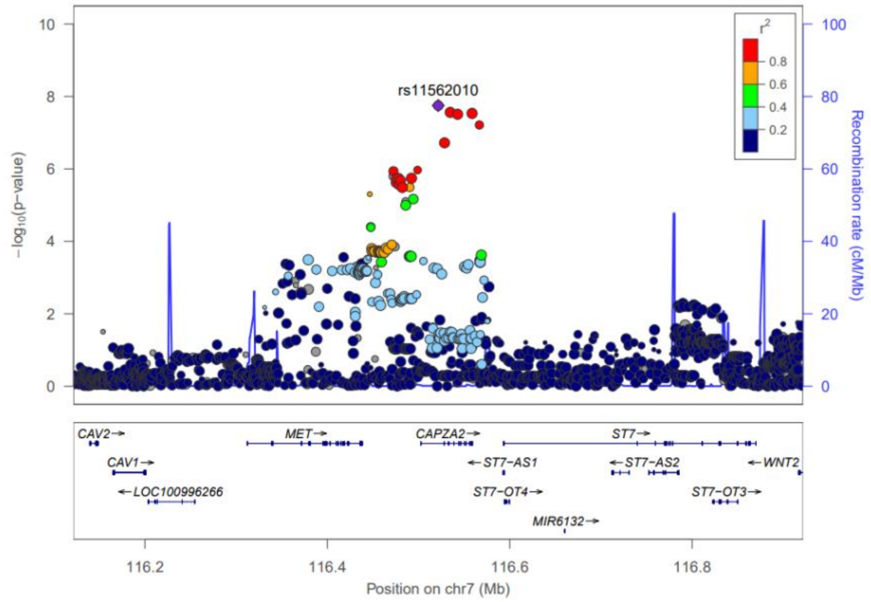

7q31.2: *CAPZA2*. We associated SNPs within *CAPZA2* (capping actin protein of muscle Z-line subunit alpha 2) with 2 ILDs and the most significant one is the distance between right endocanthion and uppermost landmark of left face contour.

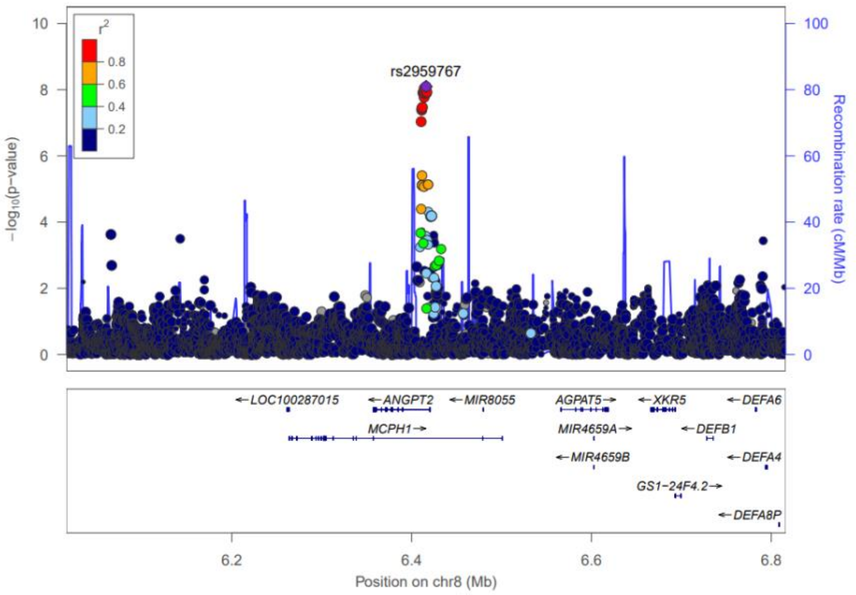

8p23.1: *MCPH1*. We associated SNPs within *MCPH1* (microcephalin 1) with four ILDs, and the most significant is the distance between the right alare and the upmost landmark of the left face contour. Mutations in *MCPH1* have been associated with primary autosomal recessive microcephaly in humans characterized by a significantly small head and brain size and intellectual disability. *Mcph1*-deficient mice were also demonstrated to have normal skull structure but smaller skull size than wild type [30]. A study identified a homozygous mutation in *MCPH1* in a microcephaly patient who also has characteristics of bird-like facies with micrognathia, craniosynostosis and ptosis [31]. One associated trait in

this study is the distance between subnasal and gnathion, which slightly corresponds to the symptom of micrognathia of the above-mentioned microcephaly patient.

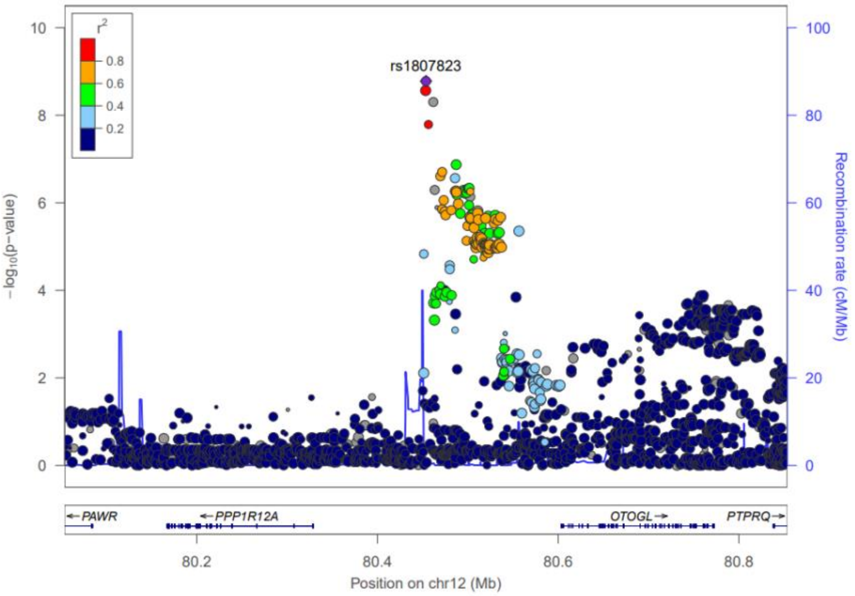

12q21.31: Intergenic (*PPP1R12A, OTOGL*). We associated SNPs from 12q21.31 region with 8 ILDs, and the most significant one is the distance between palpebrale superious and alare. Heterozygosity mutation in *PPP1R12A* (protein phosphatase 1 regulatory subunit 12A) was identified in a genitourinary and brain malformation patient [32], who also had characteristics of minor facial dysmorphisms, such as low-set ears and micrognathia (OMIM #602021). The associated traits identified in this study were all around eye and nose areas.

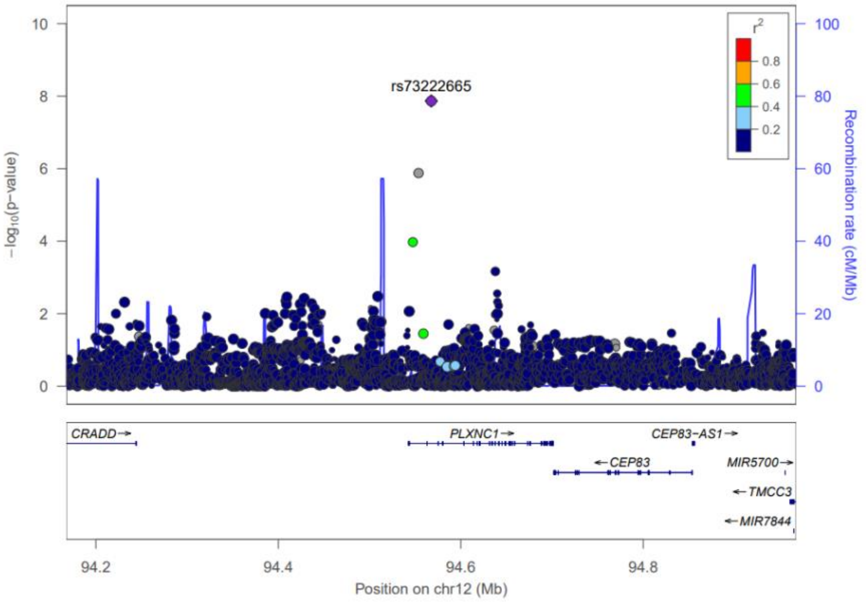

12q22: *PLXNC1*. We associated one SNP within *PLXNC1* (plexin C1) with the lower lip thickness.

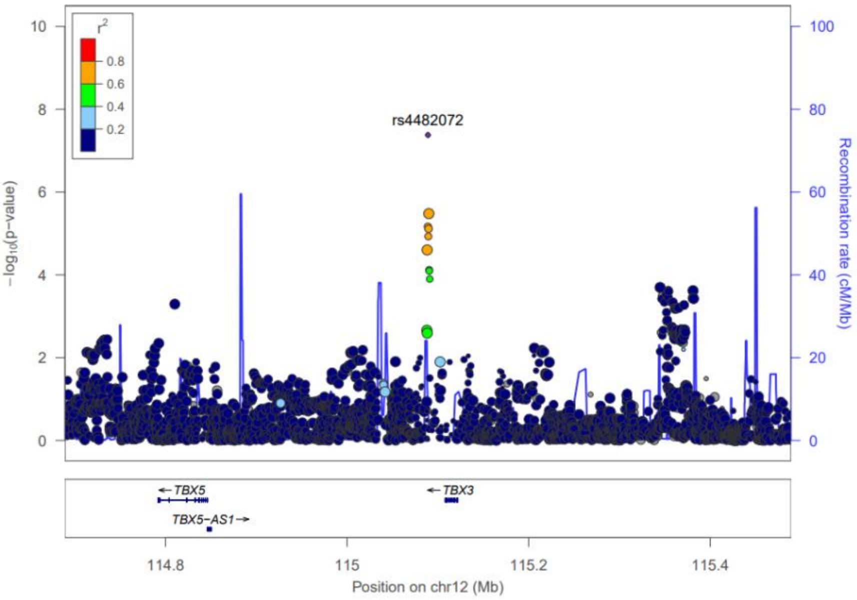

12q24.21: Intergenic (*TBX5*, *TBX3*). We associated an SNP in the intergenic region between *TBX5* (T-box transcription factor 5) and *TBX3* (T-box transcription factor 3) with the distance between right palpebrale superious and right eyebrow lower-left corner. *TBX3* has been reported to be associated with the distance between pronasale and right alare previously[10]. However, according to a conditioning analysis, our signal is independent of the ones reported before.

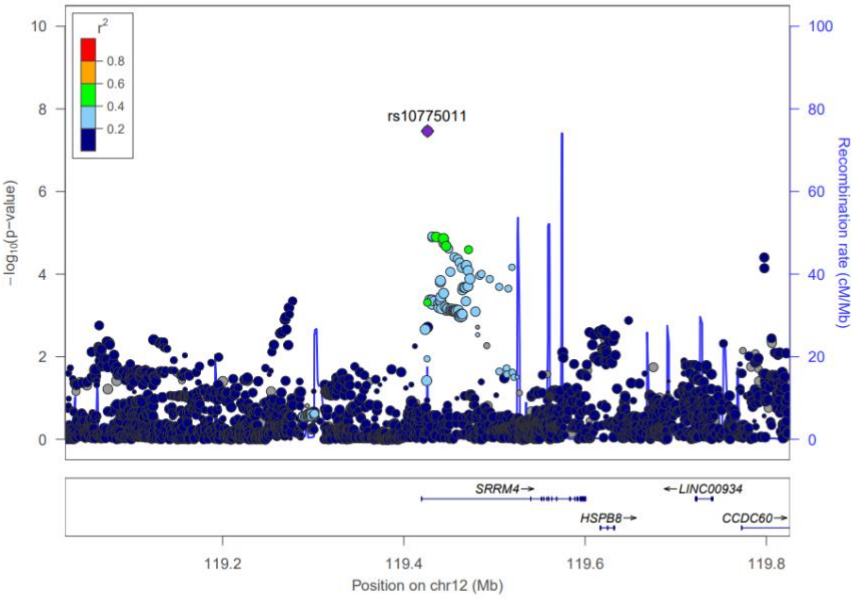

12q24.23: *SRRM4*. We associated an SNP within *SRRM4* (serine/arginine repetitive matrix 4) with the distance between the right endocanthion and the right cheilion. *Srrm4* knockdown partially restores the ability of zebrafish facial branchiomotor neurons to migrate in *rest* (RE1-silencing transcription factor) mutants. In addition, *SRRM4* has been reported to be associated with lip morphology[33].

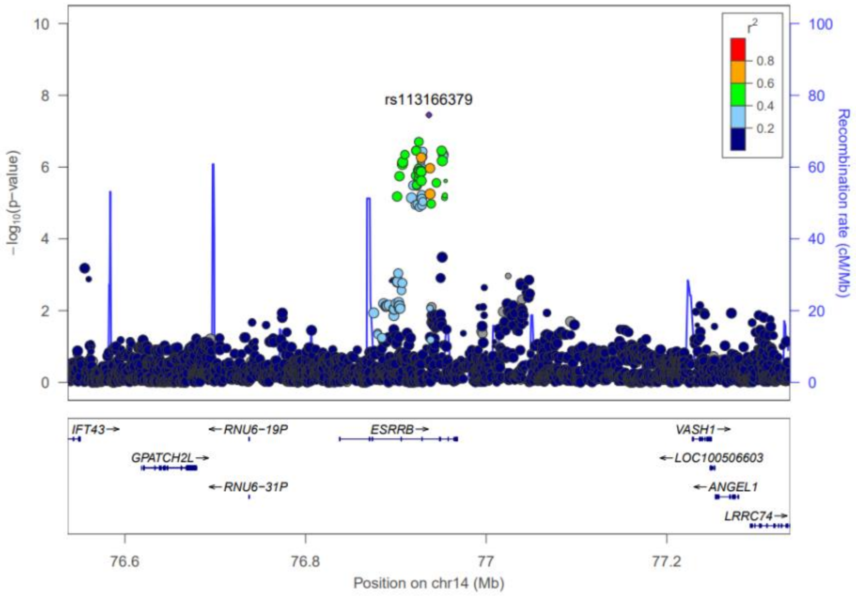

14q24.3: *ESRRB*. We associated SNPs within *ESRRB* (estrogen related receptor beta) gene with two ILDs, and the most significant one is the distance between right cheilion and the right eyebrow lower-left corner. In mice, *Esrrb* plays an essential role in placental development and is expressed during inner ear development, which indicates its essential role in inner ear development and function (OMIM #602167).

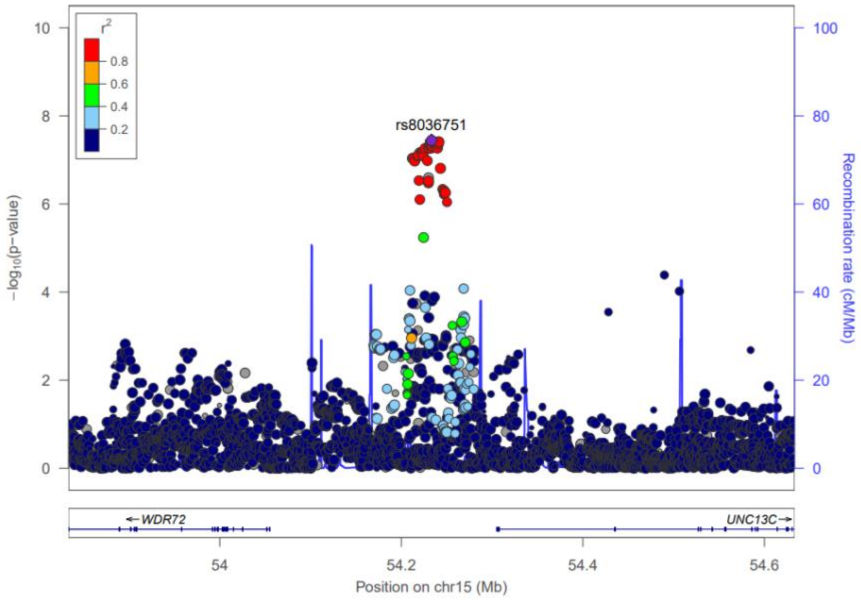

15q21.3: Intergenic (*WDR72, UNC13C*). We associated SNPs in the intergenic region between *WDR72* (WD repeat domain 72) and *UNC13C* (unc-13 homolog C) with the distance between crista philtre and stomion.

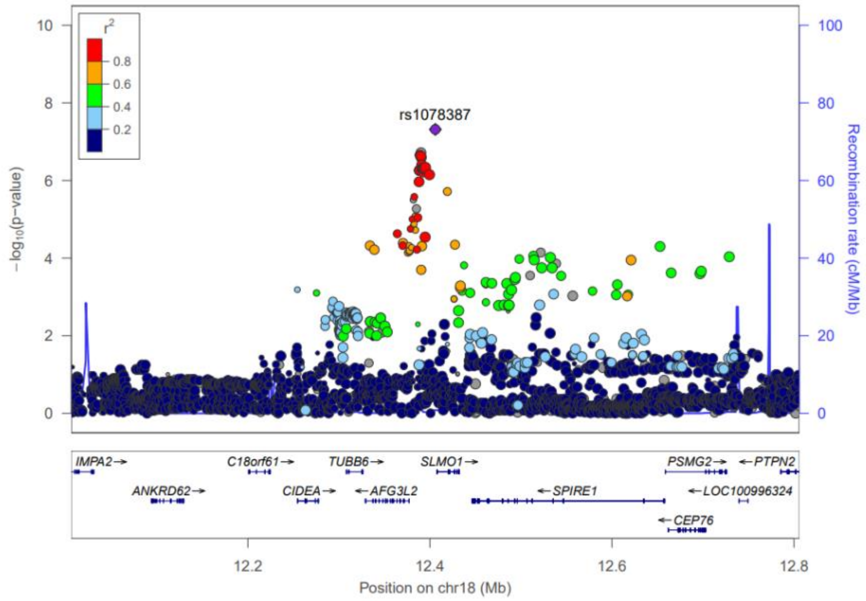

18p11.21: *TUBB6*, *SLMO1*. We associated an SNP within *SLMO1* (PRELI domain containing 3A) associated with the distance between stomion and gnathion. A previous study showed that mutations in its nearby gene *TUBB6* (tubulin beta 6 class V) would cause congenital non-progressive facial palsy[34].

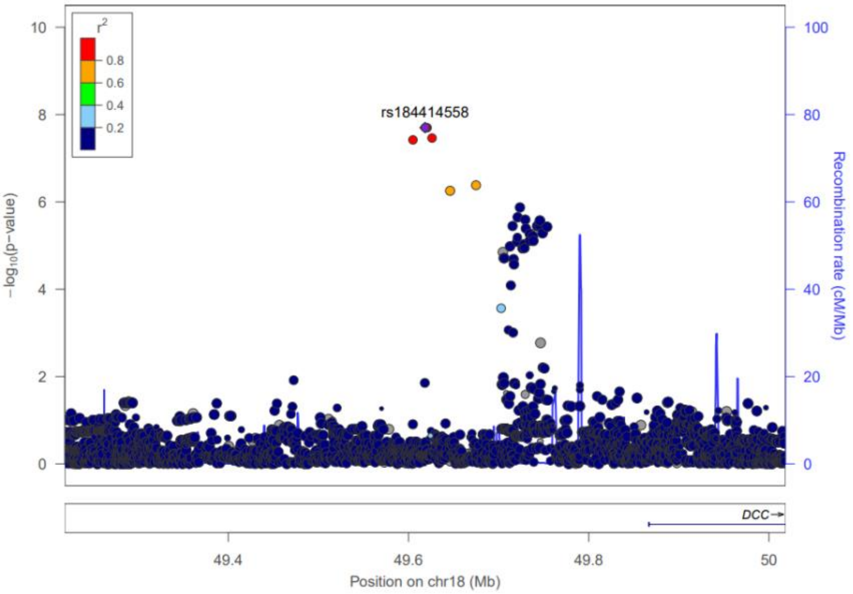

18q21.2: Intergenic (*DCC*). We associated SNPs in 18q21.2 region with two ILDs, and the most significant one is the distance between right frontotemporale and labiale inferious. *DCC* was reported to be associated with BMI[35].

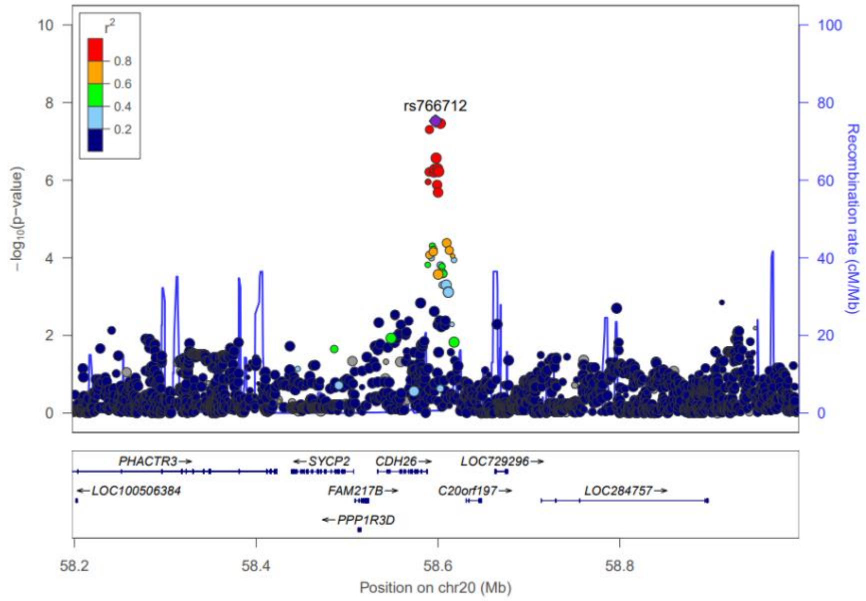

20q13.33: Intergenic (*SYCP2, CDH26, LOC729296*). We associated SNPs from 20q13.33 region with the distance between gnathion and nasion. A previous study has reported that *SYCP2* (synaptonemal complex protein 2) gene was overexpressed in Immunodeficiency, centromeric instability and facial anomalies syndrome (ICF) compared to healthy control[36].

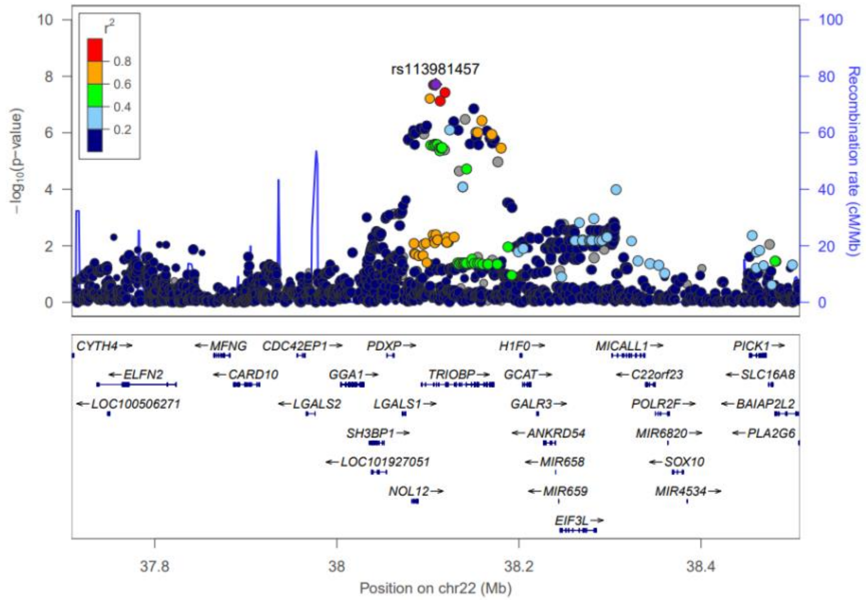

22q13.1: *NOL12, TRIOBP*. We associated SNPs within *TRIOBP* (TRIO and F-actin binding protein) genes with the distance between subnasal and right nose contour. Mutations in *TRIOBP* have been associated with a form of autosomal recessive nonsyndromic deafness[37].

Supplementary Note 3: Regional association plots for novel regions detected in this study whose replication P-values are insignificant in both Chinese and European cohorts.

It is due to either the unavailability of the SNPs in these two cohorts (first five) or weak P-value (the last one).
One genomic region on the sex chromosome (Xp22.13) could not be shown here, because locuszoom plots for X chromosome is not available.

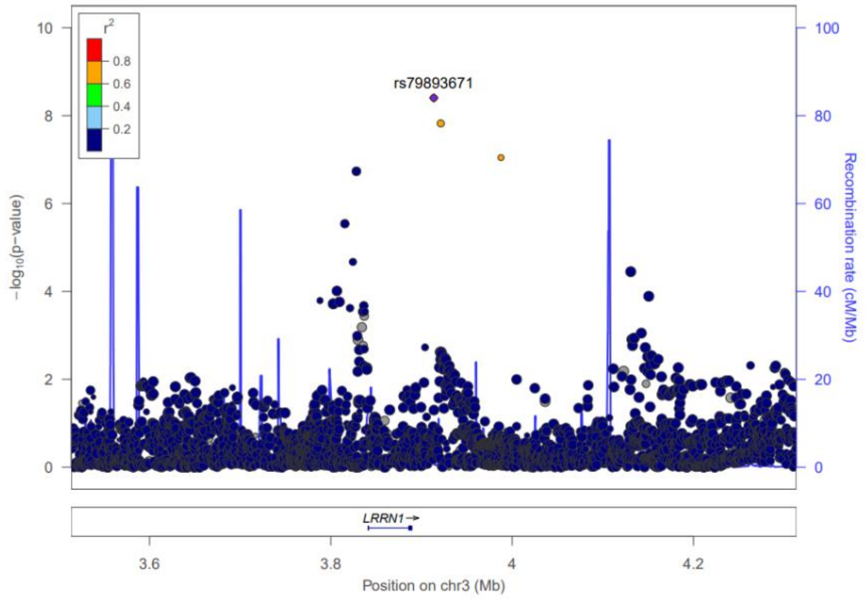

3p26.2: Intergenic (*LRRN1*). We associated SNPs in 3p26.2 region with the distance between the right alare and the upmost landmark of face contour. Mutations of several candidate genes for autism including *LRRN1* (leucine rich repeat neuronal 1) were identified in a child who also had facial dysmorphology[38].

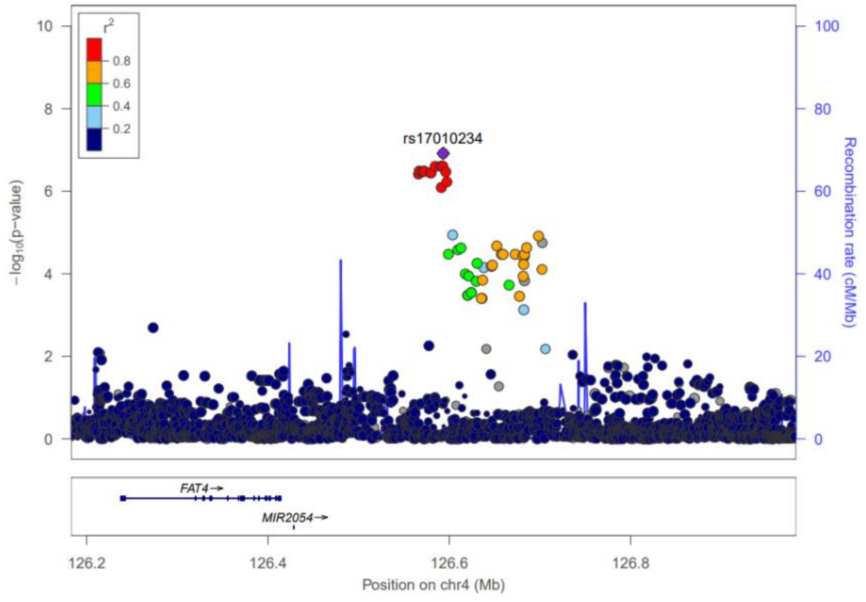

4q28.1: Intergenic (*MIR2054*). We associated an SNP in 4q28.1 region with the distance between palpebrale superious and nasal root contour.

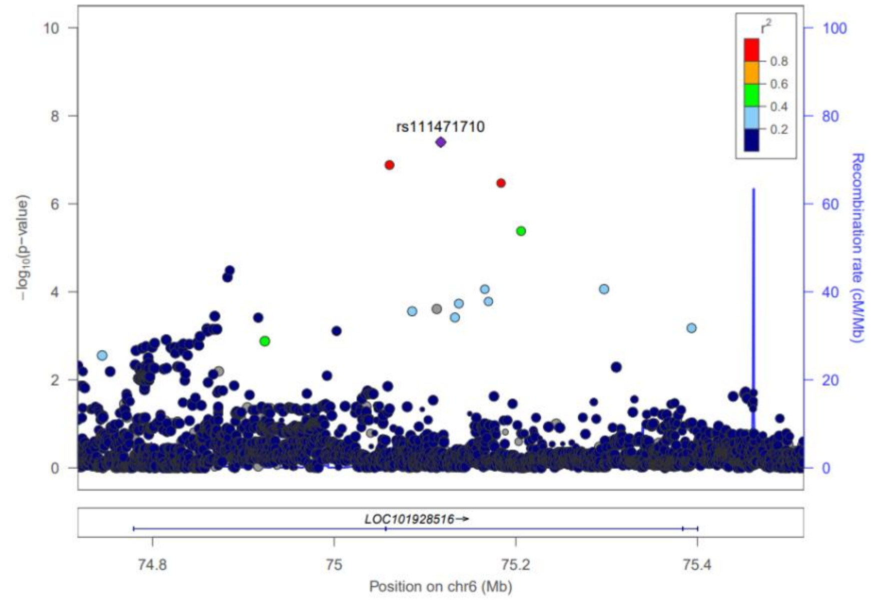

6q13: *LOC101928516*. We associated an SNP within *LOC101928561* with a distance representing eyebrow thickness.

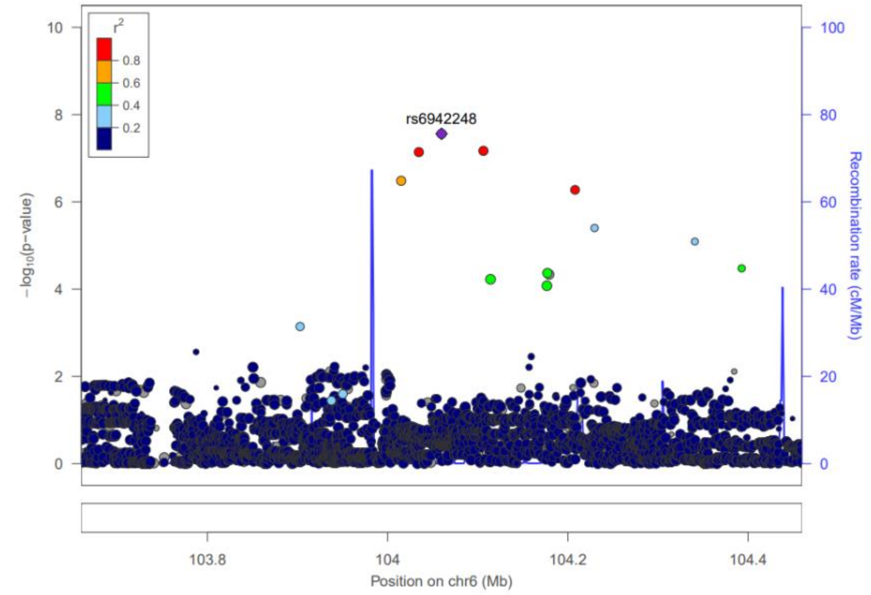

6q16.3: Intergenic. We associated an SNP in 6q16.3 region with the distance between stomion and the uppermost landmark of the right face contour.

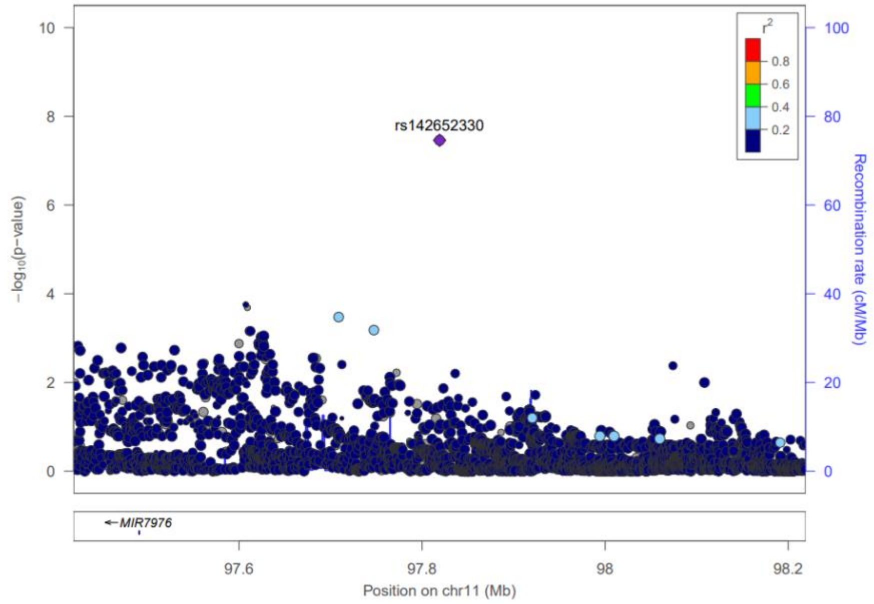

11q22.1: Intergenic (*MIR7976*). We associated one SNP in 11q22.1 region with upper lip thickness.

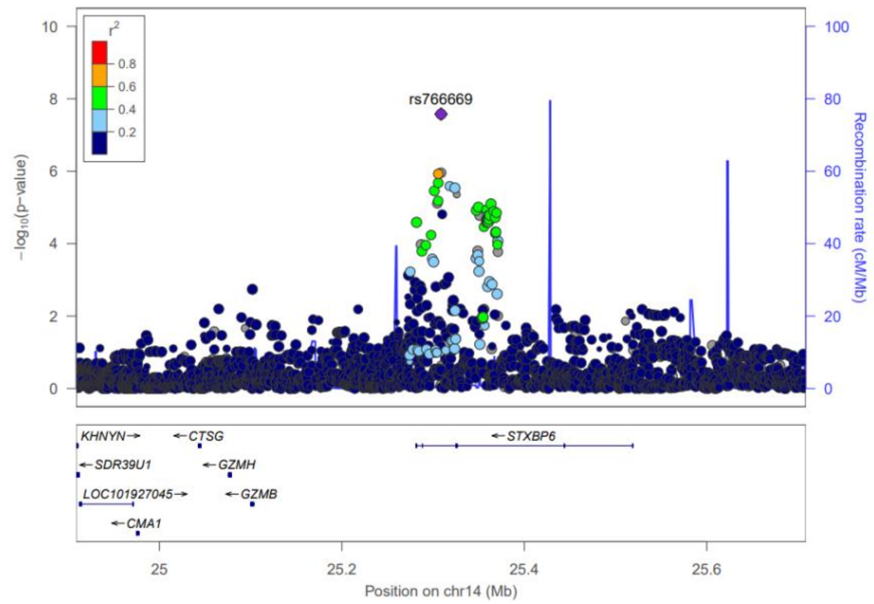

14q12: *STXBP6*. We associated an SNP within *STXBP6* (syntaxin binding protein 6) with the distance between right nose contour and right eyebrow upper left corner. Some potential regulatory pathways of *Stxbp6* in the central nervous system were suggested in the previous study[39].

Supplementary Figures

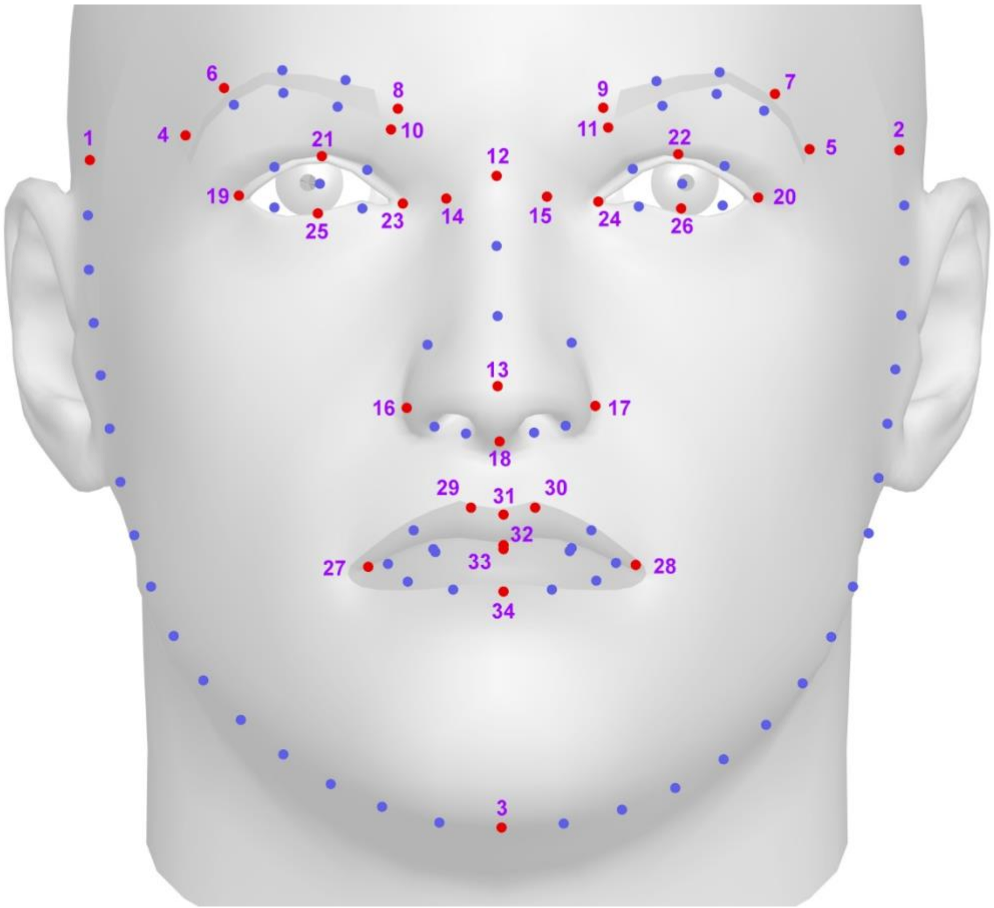

Supplementary Figure 1: Full Face++ landmarking protocol.
All the 106 landmarks are shown, and the 34 landmarks that are used to derive the 301 facial ILDs are marked in red color.

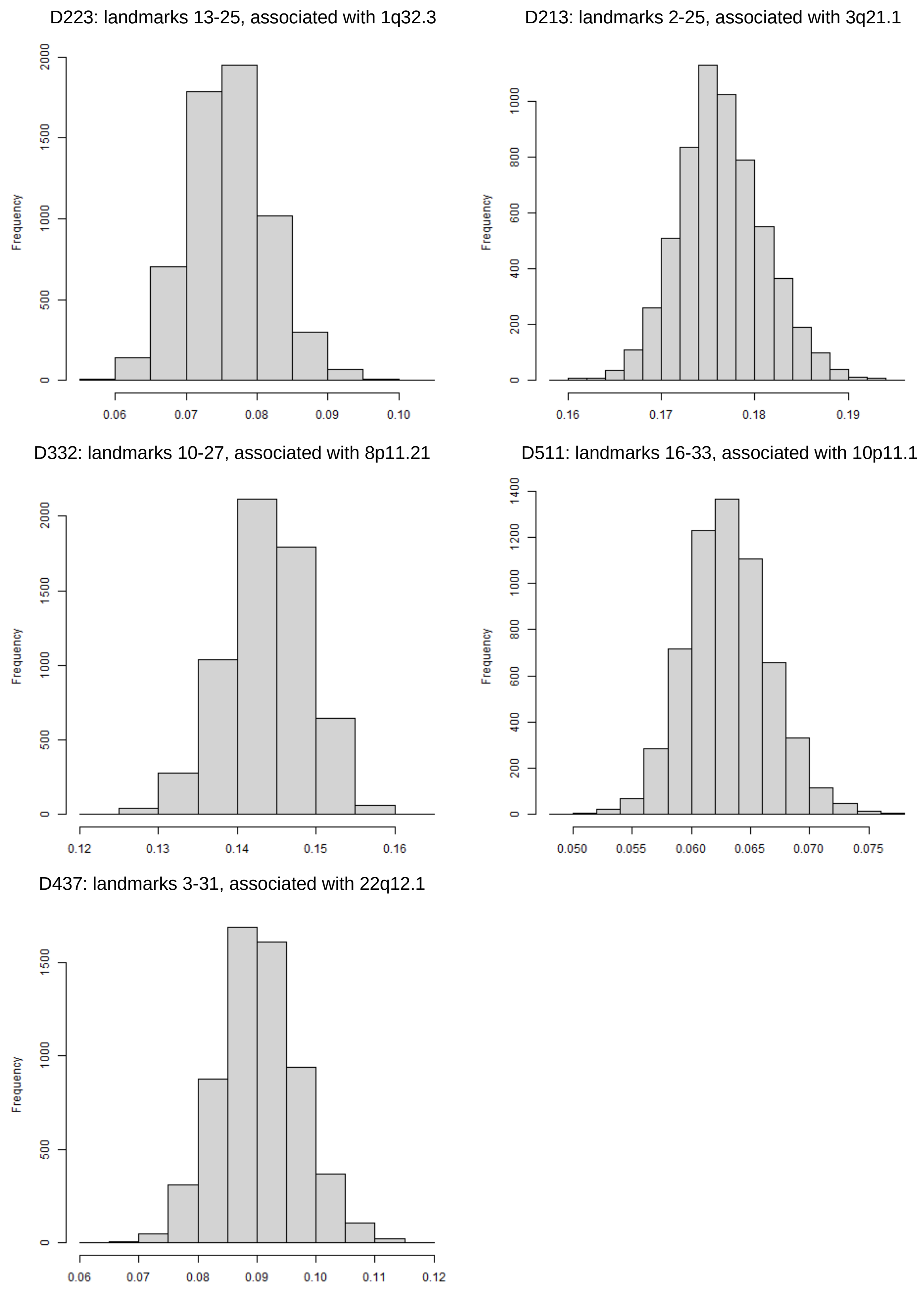

Supplementary Figure 2: Histograms for 5 Inter-landmark distances that are most significantly associated with 5 the novel genomic regions in Table 2.

Histograms for D223, D213, D332, D511 and D437. They all show considerable variation and are approximately normally distributed.

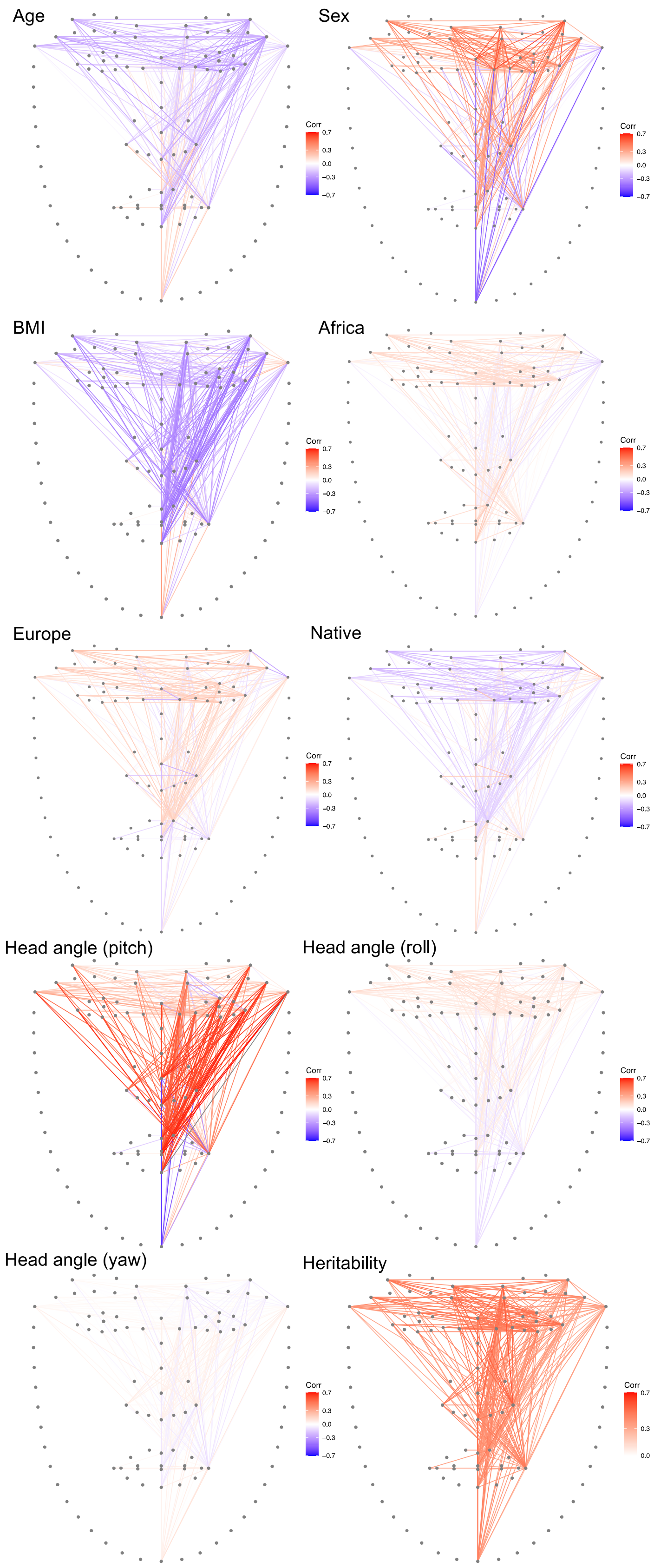

Supplementary Figure 3: Covariates correlations and heritability.
The correlation between each ILD with nine covariates (age, BMI, sex, three ancestries, and three head angles), and heritability. Red color represents a positive correlation, while blue represents a negative correlation. The darker the color, the stronger the correlation.

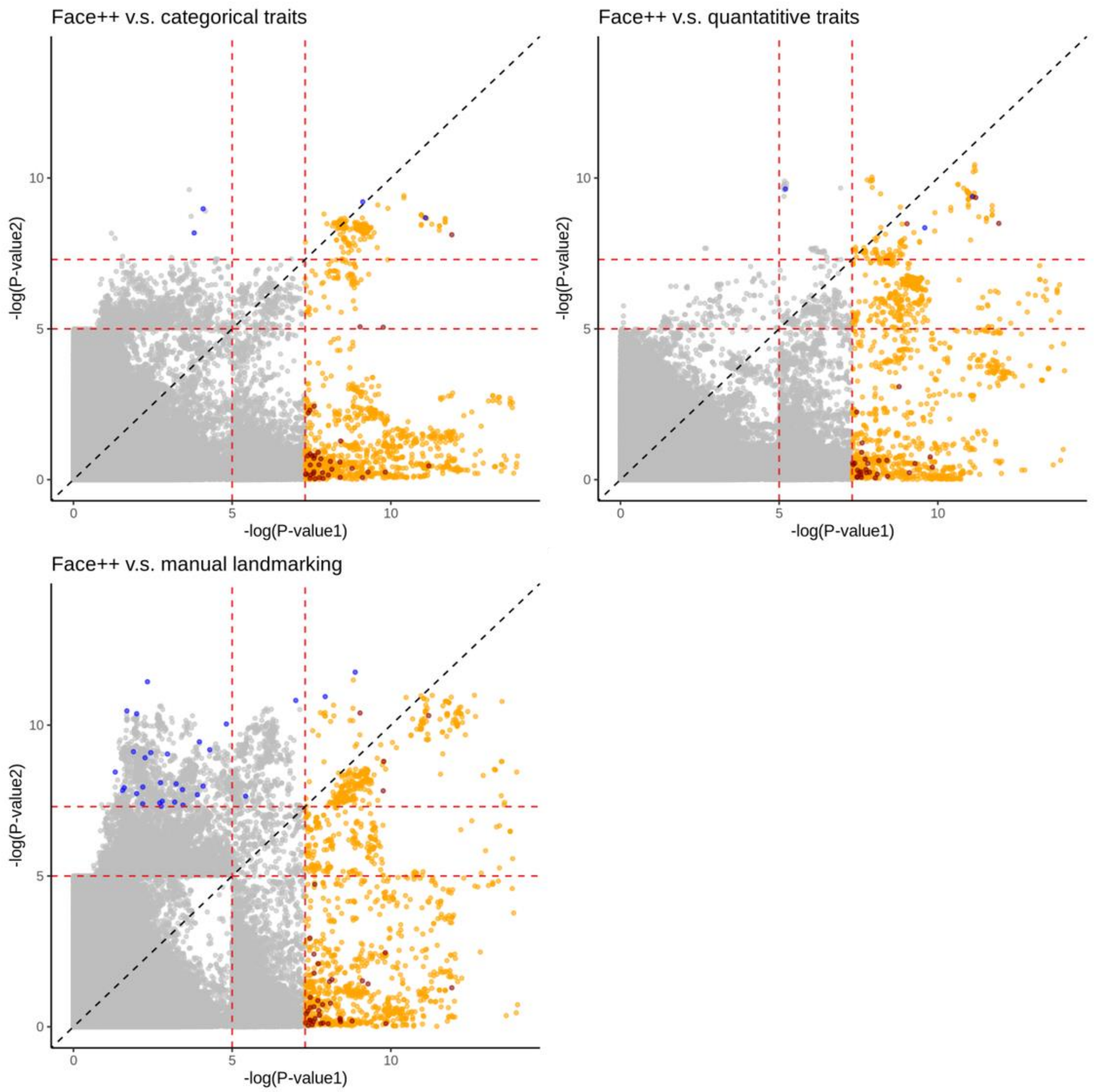

Supplementary Figure 4: Comparison of the results of this current study with three previous analyses of the same cohort.

We compared the results of this study with those of three previous analyses using different phenotyping protocols. The results of the two datasets, one using categorical phenotyping and the other using quantitative traits, were published in Adhikari et al., 2016[1]. Another study using measurements derived from a manual landmarking protocol on profile photographs was published in Bonfante et al., 2021[2].

In the scatterplot, we selected the lowest P-values across all examined traits of each SNP. Each dot in the graph represents a single SNP. X-axis represents the -logP-value of this study, and Y-axis represents the -logP-value of previous studies. The red dotted lines in the graph represent the suggestive significant threshold (-logP=5), and the genome-wide significant threshold (-logP=7.3). The black dotted line is the diagonal line. General SNPs are plotted in grey, genome-wide significant SNPs in this study are plotted in orange, index SNPs in this study are plotted in red, and index SNPs in the previous studies are plotted in blue.

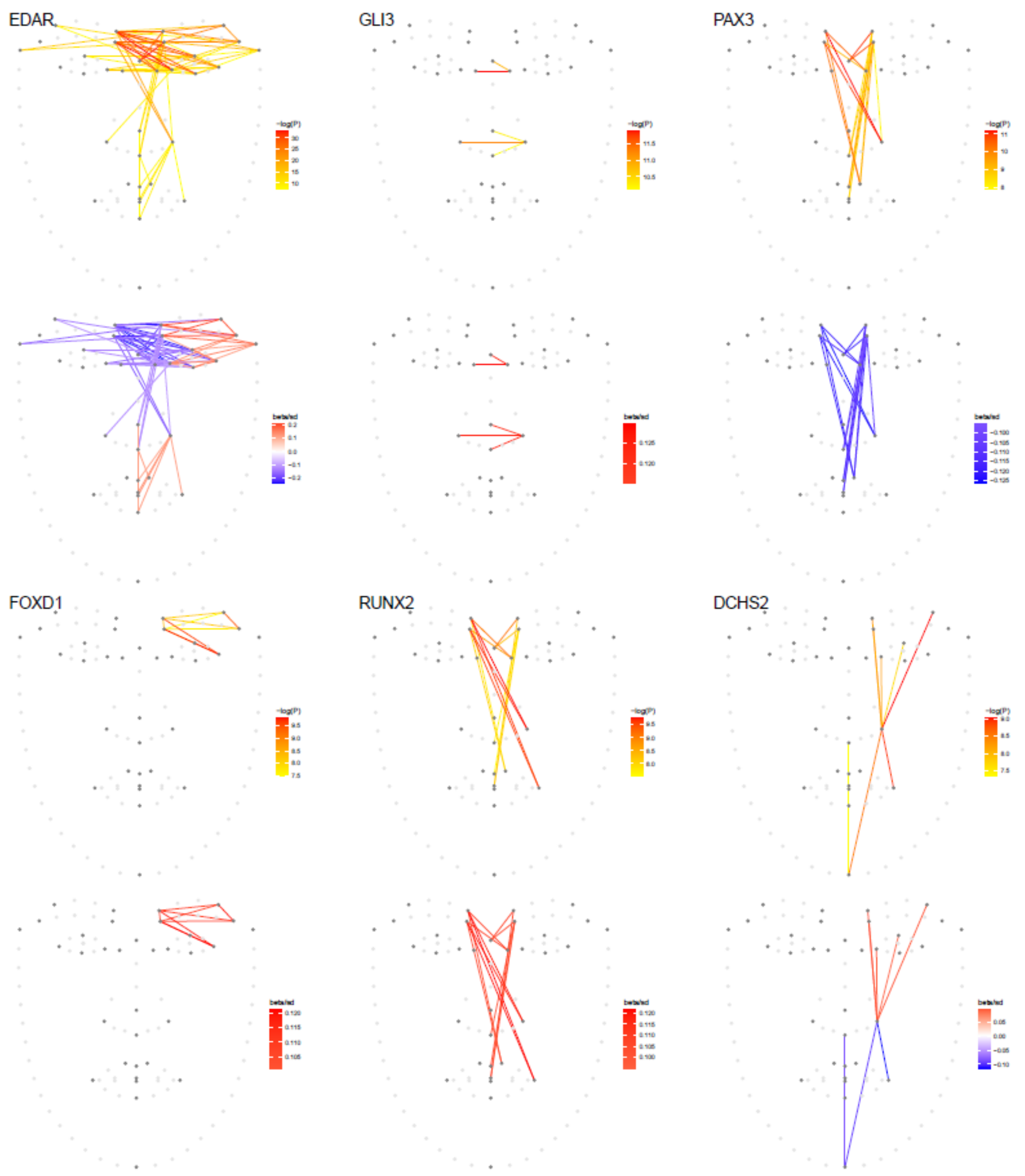

Supplementary Figure 5: Illustration of inter-landmark distances associated with the 6 most robustly replicated signals (*EDAR*, *GLI3*, *PAX3*, *FOXD1*, *RUNX2*, *DCHS2*).
Red/yellow color gradient reflects the strength of SNP association with a facial distance. Red/blue gradient indicates the direction of the SNP effect.

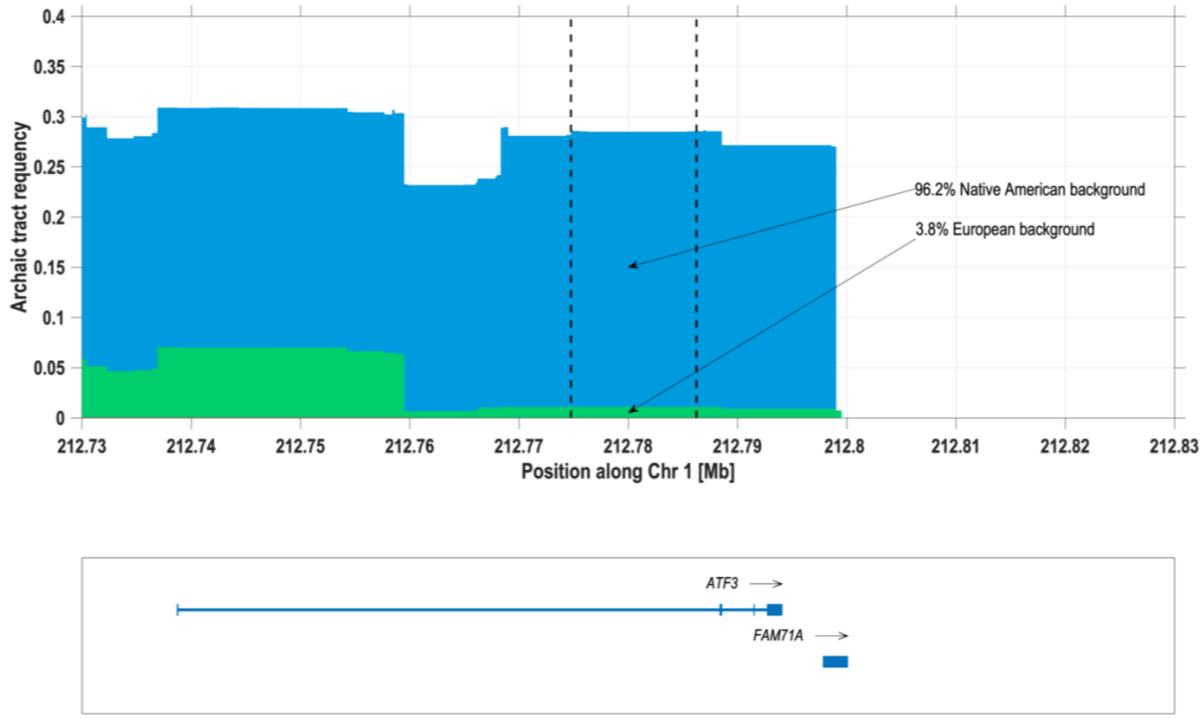

Supplementary Figure 6: Continental ancestry proportions in archaic introgression near *ATF3*.

The panel below features the cumulated frequency distribution of Neanderthal introgressed segments near *ATF3* (1q32.3), hence identical to the middle panel of Figure 4 except that it was here painted in accordance to the continental ancestry called on the same chromosomes (green = European, blue = Native American).

The dashed vertical lines denote the limits of the most significant segment (which also overlaps regulatory elements – see Figure 4). On average over that portion, 96.2% of introgressed segments are found on tracts of Native American ancestry. Local ancestry estimates were previously obtained using RFMix (v1). The proportion of introgressed segments found on a Native American background drops to 83.1% when considering the whole region shown in this plot (100 Kb).

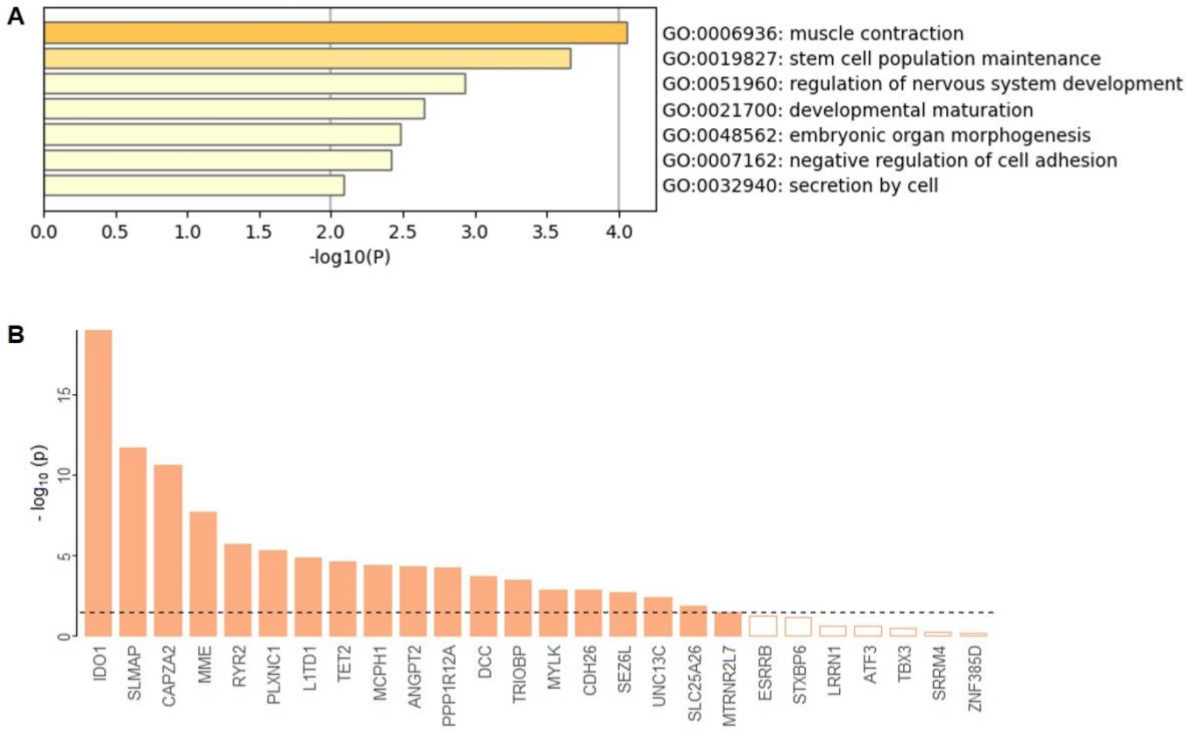

Supplementary Figure 7: GO enrichment analysis and comparison of expression levels for transcripts nearest to the novel index SNPs.

A. Metascape enrichment analysis of all nearest genes to the index SNPs (see Supplementary Table 4) in Gene Ontology biological processes.

B. Comparison of transcription levels (normalized RNA-seq VST values) in cranial neural crest cells (CNCCs; Prescott et al. 2015[40]), relative to cells from the ENCODE database (ENCODE Project Consortium, 2012[41]). The dotted line indicates a Benjamini-Hochberg's FDR threshold for significant difference in transcription levels ($p<0.0340$). Significant differences between CNCC v. Encode expression levels are also highlighted with filled boxes.

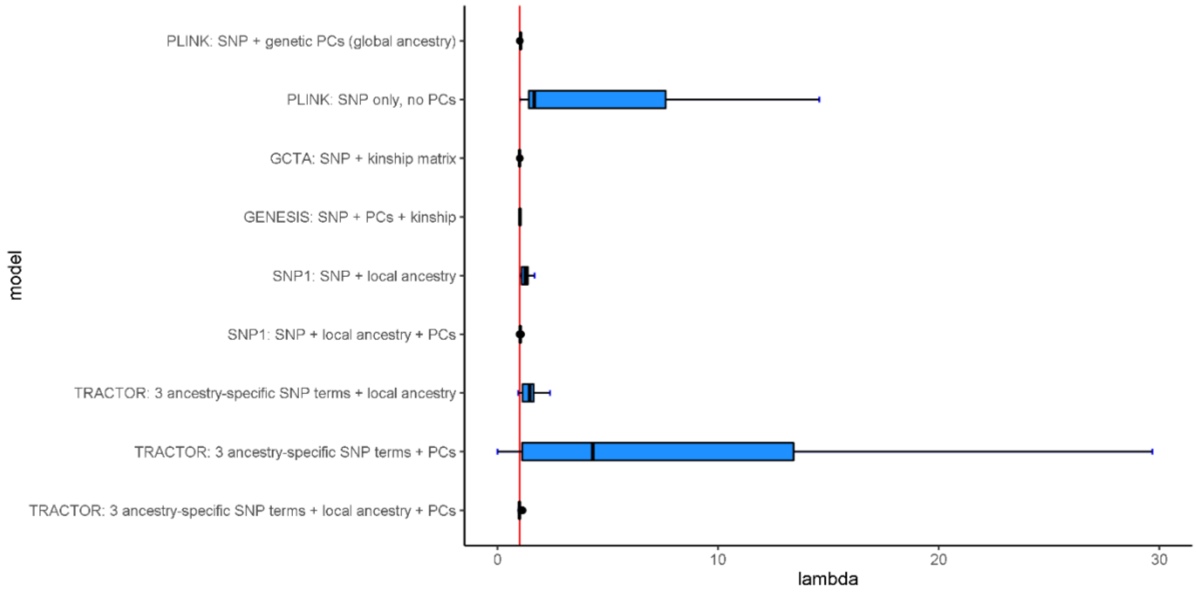

Supplementary Figure 8: Boxplot of lambda values for GWASs of 148 facial distances based on nine different analysis models, using PLINK, GCTA, GENESIS, TRACTOR, or SNP1.

The red line represents lambda = 1 (i.e. no inflation). The X axis is truncated at 30. The median and maximum lambda values for each of these models are reported in Supplementary Table 8.

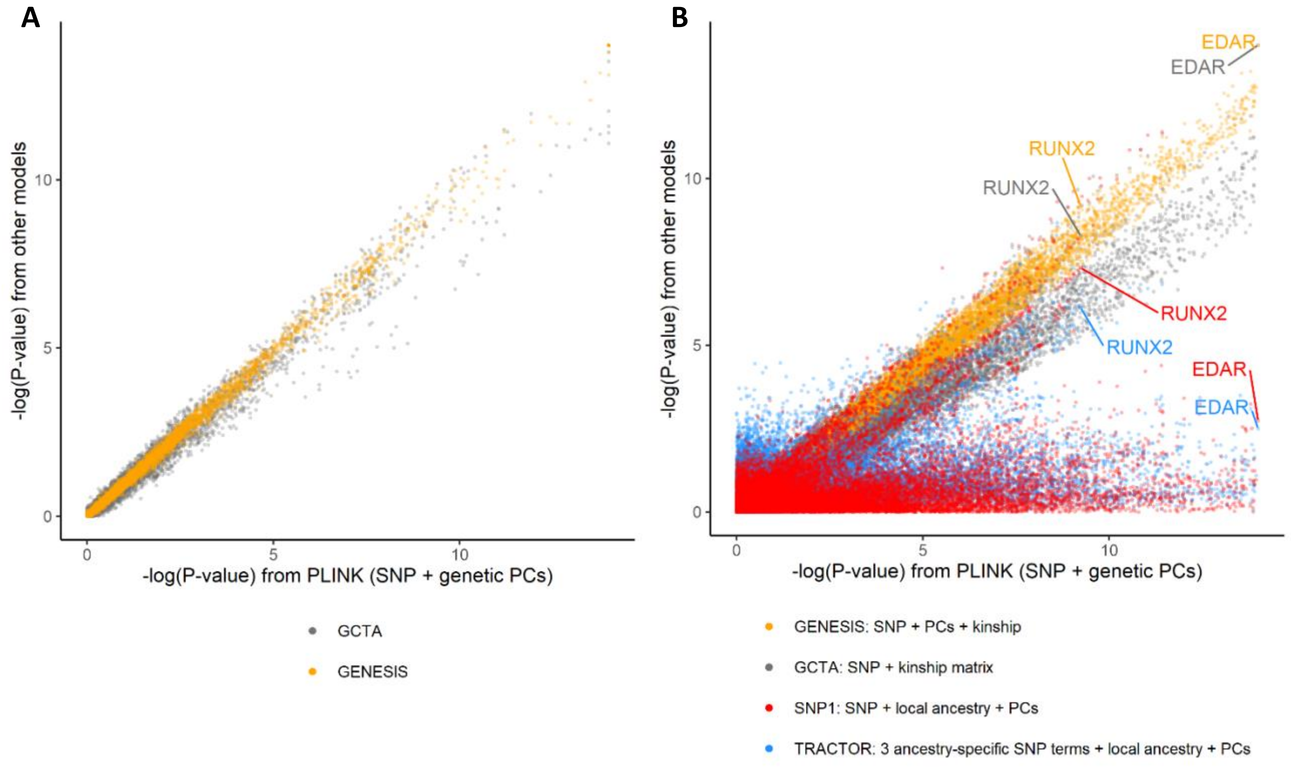

Supplementary Figure 9: Comparison of SNP -log(P-values) obtained by PLINK with values obtained using GCTA GENESIS, TRACTOR, and SNP1 (across 148 associated distances).

(A) Scatter plot of the -log(P-values) for 42 index SNPs (genotyped and imputed) obtained with PLINK (X-axis) compared to values for these SNPs obtained with GENESIS (orange) or GCTA (grey). Axes are truncated at 14. (B) Scatterplot of the -log(P-values) for 151 chip-genotyped SNPs obtained with PLINK (X-axis), compared to the values obtained with GENESIS (orange), GCTA (grey), TRACTOR (blue) and SNP1 (red). Axes are truncated at 14. The most significant SNPs for two well-established genes (*EDAR* and *RUNX2*) are indicated.

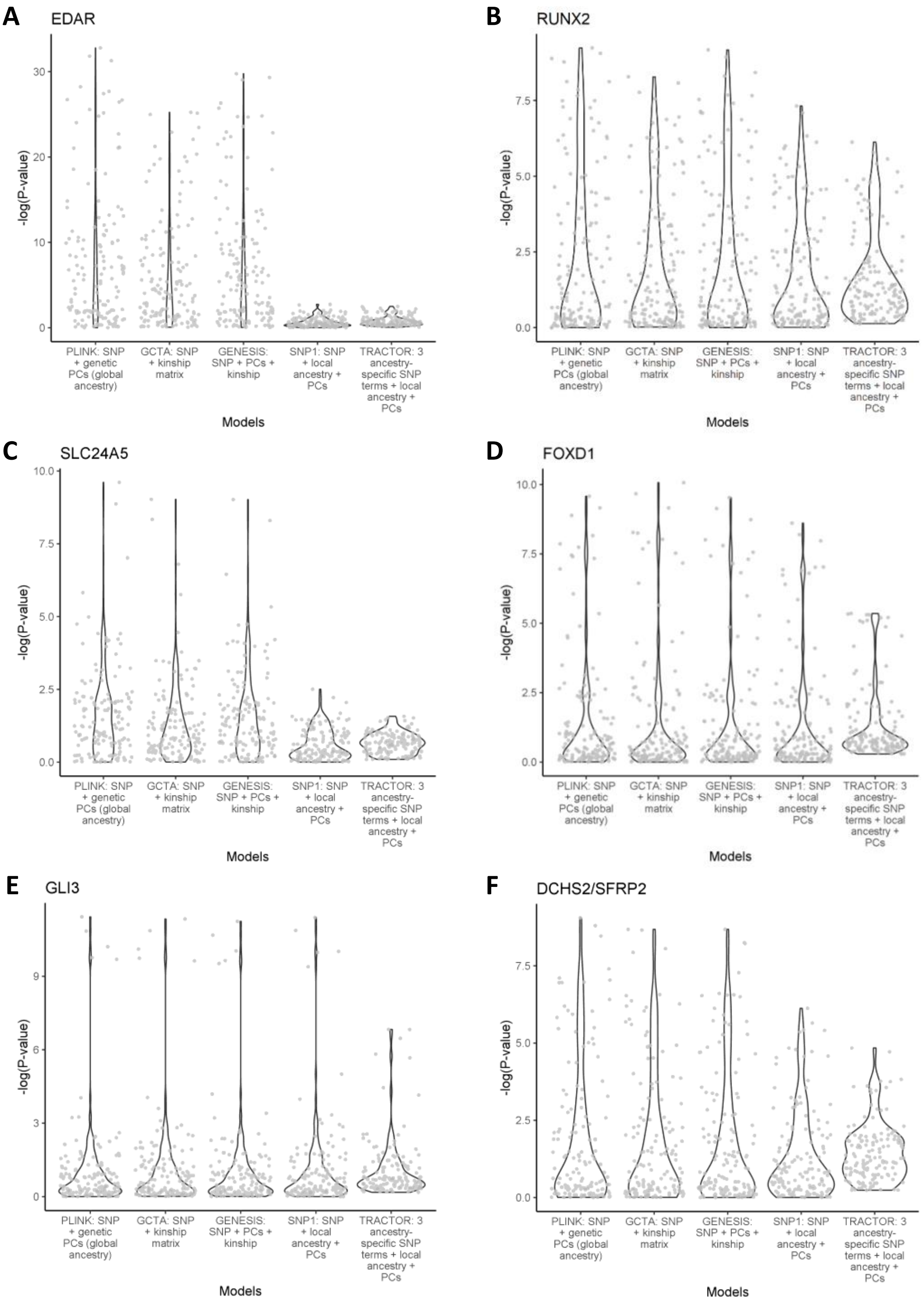

Supplementary Figure 10: Violin plots of -log(P-values) obtained using PLINK, GCTA, GENESIS, TRACTOR, and SNP1 for index SNPs at six well-established face gene regions (Table 1) across 148 facial distances. For *SLC24A5* and *DCHS2* the plot displays values for the genotyped index SNP shown in Table 1 (the one with smallest P-value across traits). For the other genes the index SNP of Table 1 was imputed and we selected the chip-genotyped SNP with smallest P-value across traits amongst SNPs in high LD with the index SNP of Table 1 (r^2>0.9). For the other three gene regions shown in Table 1 there were no genotyped SNPs that were in LD with the imputed index SNP and showed significant association.

Supplementary Tables

Supplementary Table 1: Interclass correlation coefficient and median distance for each corresponding landmark pair from Face++, Dlib and manual protocols.

Supplementary Table 2: Description of the 34 well-defined Face++ landmarks grouped by regions that used in the inter-landmarks distances calculation.

Supplementary Table 3: Covariates correlations and narrow-sense heritabilities.

Supplementary Table 4: Features of the regions showing genome-wide significant association in this study.

Supplementary Table 5: Follow-up of the 33 novel face loci in the mouse.

Supplementary Table 6: Features of tested admixture mapping segments in this study.

Supplementary Table 7: Nasal height (NLH) in Neanderthal and three modern human populations.

Supplementary Table 8: Summary of GWAS associated regions between FDR threshold (1.82×10^{-6}) and nominal GWAS threshold (5×10^{-8}).

Supplementary Table 9: Summary of condition analysis to determine if the signal is reported previously.

Supplementary Table 10: Summary of genomic inflation factor (lambda) from various GWAS models.

Supplementary Table 11: Cell type classification of RNA-seq samples from ENCODE used in study.

Supplementary Tables can be found in the Supplementary Data file.

Supplementary Movie: Mouse craniofacial morphology impacted by 22q12.1 (index SNP: rs32069343).

Supplementary movie can be found in Supplementary_movie.mp4

References

1. Adhikari, K., et al., *A genome-wide association scan implicates DCHS2, RUNX2, GLI3, PAX1 and EDAR in human facial variation.* Nature Communications, 2016. **7**.
2. Bonfante, B., et al., *A GWAS in Latin Americans identifies novel face shape loci, implicating VPS13B and a Denisovan introgressed region in facial variation.* Science Advances, 2021. **7**(6).
3. Li, Y., et al., *EDAR, LYPLAL1, PRDM16, PAX3, DKK1, TNFSF12, CACNA2D3, and SUPT3H gene variants influence facial morphology in a Eurasian population.* Human Genetics, 2019. **138**(6): p. 681-689.
4. Adhikari, K., et al., *A genome-wide association scan in admixed Latin Americans identifies loci influencing facial and scalp hair features.* Nature Communications, 2016. **7**.
5. Claes, P., et al., *Genome-wide mapping of global-to-local genetic effects on human facial shape.* Nature Genetics, 2018. **50**(3): p. 414-+.
6. Liu, F., et al., *A genome-wide association study identifies five loci influencing facial morphology in Europeans.* PLoS Genet, 2012. **8**(9): p. e1002932.
7. Paternoster, L., et al., *Genome-wide association study of three-dimensional facial morphology identifies a variant in PAX3 associated with nasion position.* Am J Hum Genet, 2012. **90**(3): p. 478-85.
8. Pickrell, J.K., et al., *Detection and interpretation of shared genetic influences on 42 human traits (vol 48, pg 709, 2016).* Nature Genetics, 2016. **48**(10): p. 1296-1296.
9. White, J.D., et al., *Insights into the genetic architecture of the human face.* Nature Genetics, 2021. **53**(1).
10. Xiong, Z.Y., et al., *Novel genetic loci affecting facial shape variation in humans.* Elife, 2019. **8**.
11. Boudjadi, S., et al., *The expression and function of PAX3 in development and disease.* Gene, 2018. **666**: p. 145-157.
12. Kurosaka, H., et al., *Disrupting hedgehog and WNT signaling interactions promotes cleft lip pathogenesis.* J Clin Invest, 2014. **124**(4): p. 1660-71.
13. Le Pabic, P., C. Ng, and T.F. Schilling, *Fat-Dachsous signaling coordinates cartilage differentiation and polarity during craniofacial development.* PLoS Genet, 2014. **10**(10): p. e1004726.
14. Milunsky, J.M., et al., *LADD syndrome is caused by FGF10 mutations.* Clinical Genetics, 2006. **69**(4): p. 349-354.
15. Wu, S.J., et al., *Genome-wide association studies and CRISPR/Cas9-mediated gene editing identify regulatory variants influencing eyebrow thickness in humans.* Plos Genetics, 2018. **14**(9).
16. Sennett, R., et al., *An Integrated Transcriptome Atlas of Embryonic Hair Follicle Progenitors, Their Niche, and the Developing Skin.* Developmental Cell, 2015. **34**(5): p. 577-591.
17. Sears, K.E., et al., *The correlated evolution of Runx2 tandem repeats, transcriptional activity, and facial length in Carnivora.* Evolution & Development, 2007. **9**(6): p. 555-565.
18. Komori, T., *Roles of Runx2 in Skeletal Development.* Adv Exp Med Biol, 2017. **962**: p. 83-93.
19. Otto, F., et al., *Cbfa1, a candidate gene for cleidocranial dysplasia syndrome, is essential for osteoblast differentiation and bone development.* Cell, 1997. **89**(5): p. 765-771.
20. Komori, T., et al., *Targeted disruption of Cbfa1 results in a complete lack of bone formation owing to maturational arrest of osteoblasts.* Cell, 1997. **89**(5): p. 755-764.

21. Purandare, S.M., et al., *De novo three-way chromosome translocation 46,XY,t(4;6;21)(p16;p21.1;q21) in a male with cleidocranial dysplasia.* American Journal of Medical Genetics Part A, 2008. **146a**(4): p. 453-458.
22. Ritter, D.I., et al., *Identifying gene disruptions in novel balanced de novo constitutional translocations in childhood cancer patients by whole-genome sequencing.* Genetics in Medicine, 2015. **17**(10): p. 831-835.
23. Marigo, V., et al., *Sonic hedgehog differentially regulates expression of GLI and GLI3 during limb development.* Developmental Biology, 1996. **180**(1): p. 273-283.
24. Abdullah, et al., *Variants in GLI3 Cause Greig Cephalopolysyndactyly Syndrome.* Genetic Testing and Molecular Biomarkers, 2019. **23**(10): p. 744-750.
25. Roscioli, T., et al., *Pallister-Hall syndrome: Unreported skeletal features of a GLI3 mutation.* American Journal of Medical Genetics Part A, 2005. **136a**(4): p. 390-394.
26. Zhou, X., et al., *Genome-wide CRISPR knockout screens identify ADAMTSL3 and PTEN genes as suppressors of HCC proliferation and metastasis, respectively.* J Cancer Res Clin Oncol, 2020. **146**(6): p. 1509-1521.
27. Jung, C.B., et al., *Dantrolene rescues arrhythmogenic RYR2 defect in a patient-specific stem cell model of catecholaminergic polymorphic ventricular tachycardia.* EMBO Mol Med, 2012. **4**(3): p. 180-91.
28. Sousa, S.B., et al., *Postnatal growth retardation, facial dysmorphism, spondylocarpal synostosis, cardiac defect, and inner ear malformation (cardiospondylocarpofacial syndrome?)--a distinct syndrome?* Am J Med Genet A, 2010. **152A**(3): p. 539-46.
29. Higuchi, Y., et al., *Mutations in MME cause an autosomal-recessive Charcot-Marie-Tooth disease type 2.* Ann Neurol, 2016. **79**(4): p. 659-72.
30. Chen, J., et al., *Mcph1-Deficient Mice Reveal a Role for MCPH1 in Otitis Media.* Plos One, 2013. **8**(3).
31. Farooq, M., et al., *Craniosynostosis-microcephaly with chromosomal breakage and other abnormalities is caused by a truncating MCPH1 mutation and is allelic to premature chromosomal condensation syndrome and primary autosomal recessive microcephaly type 1.* Am J Med Genet A, 2010. **152A**(2): p. 495-7.
32. Hughes, J.J., et al., *Loss-of-Function Variants in PPP1R12A: From Isolated Sex Reversal to Holoprosencephaly Spectrum and Urogenital Malformations.* Am J Hum Genet, 2020. **106**(1): p. 121-128.
33. Lee, M.K., et al., *Genome-wide association study of facial morphology reveals novel associations with FREM1 and PARK2.* PLoS One, 2017. **12**(4): p. e0176566.
34. Fazeli, W., et al., *A TUBB6 mutation is associated with autosomal dominant non-progressive congenital facial palsy, bilateral ptosis and velopharyngeal dysfunction.* Hum Mol Genet, 2017. **26**(20): p. 4055-4066.
35. Velez Edwards, D.R., et al., *Gene-environment interactions and obesity traits among postmenopausal African-American and Hispanic women in the Women's Health Initiative SHARe Study.* Hum Genet, 2013. **132**(3): p. 323-36.
36. Simo-Riudalbas, L., et al., *Genome-Wide DNA Methylation Analysis Identifies Novel Hypomethylated Non-Pericentromeric Genes with Potential Clinical Implications in ICF Syndrome.* PLoS One, 2015. **10**(7): p. e0132517.
37. Zaharija, B., B. Samardzija, and N.J. Bradshaw, *The TRIOBP Isoforms and Their Distinct Roles in Actin Stabilization, Deafness, Mental Illness, and Cancer.* Molecules, 2020. **25**(21).
38. Davis, L.K., et al., *Novel copy number variants in children with autism and additional developmental anomalies.* J Neurodev Disord, 2009. **1**(4): p. 292-301.
39. Liu, C., et al., *Behavioral and Gene Expression Analysis of Stxbp6-Knockout Mice.* Brain Sci, 2021. **11**(4).

40. Prescott, S.L., et al., *Enhancer Divergence and cis-Regulatory Evolution in the Human and Chimp Neural Crest.* Cell, 2015. **163**(1): p. 68-83.
41. Consortium, E.P., *An integrated encyclopedia of DNA elements in the human genome.* Nature, 2012. **489**(7414): p. 57-74.
