# Supplementary material for: Automatic landmarking identifies new loci associated with face morphology and implicates Neanderthal introgression in human nasal shape
Source: Commun Biol. 2023 May 8;6:481. doi: 10.1038/s42003-023-04838-7 (PMC10167347; doi:10.1038/s42003-023-04838-7)
Supplement: Supplementary file 3 — Description of Additional Supplementary Files [file 42003_2023_4838_MOESM3_ESM.docx]

**Description of Additional Supplementary Files**

**File name:** Supplementary Data

**Description:** The Supplementary Data file includes eleven Supplementary Tables.

**File name:** Supplementary Movie

**Description:** Mouse craniofacial morphology impacted by 22q12.1 homologous region
