# Supplementary material for: Automatic landmarking identifies new loci associated with face morphology and implicates Neanderthal introgression in human nasal shape
Source: Commun Biol. 2023 May 8;6:481. doi: 10.1038/s42003-023-04838-7 (PMC10167347; doi:10.1038/s42003-023-04838-7)
Supplement: Supplementary file 6 — Reporting Summary [file 42003_2023_4838_MOESM6_ESM.pdf]

nature research

Corresponding author(s): Kaustubh Adhikari, Andrés Ruiz-Linares

Last updated by author(s): Mar 16, 2023

Reporting Summary

Nature Research wishes to improve the reproducibility of the work that we publish. This form provides structure for consistency and transparency in reporting. For further information on Nature Research policies, see our Editorial Policies and the Editorial Policy Checklist.

Statistics

For all statistical analyses, confirm that the following items are present in the figure legend, table legend, main text, or Methods section.

n/a	Confirmed	
☐	☒	The exact sample size (n) for each experimental group/condition, given as a discrete number and unit of measurement
☐	☒	A statement on whether measurements were taken from distinct samples or whether the same sample was measured repeatedly
☐	☒	The statistical test(s) used AND whether they are one- or two-sided<br>*Only common tests should be described solely by name; describe more complex techniques in the Methods section.*
☐	☒	A description of all covariates tested
☐	☒	A description of any assumptions or corrections, such as tests of normality and adjustment for multiple comparisons
☐	☒	A full description of the statistical parameters including central tendency (e.g. means) or other basic estimates (e.g. regression coefficient) AND variation (e.g. standard deviation) or associated estimates of uncertainty (e.g. confidence intervals)
☐	☒	For null hypothesis testing, the test statistic (e.g. F, t, r) with confidence intervals, effect sizes, degrees of freedom and P value noted<br>*Give P values as exact values whenever suitable.*
☒	☐	For Bayesian analysis, information on the choice of priors and Markov chain Monte Carlo settings
☒	☐	For hierarchical and complex designs, identification of the appropriate level for tests and full reporting of outcomes
☐	☒	Estimates of effect sizes (e.g. Cohen's d, Pearson's r), indicating how they were calculated

Our web collection on statistics for biologists contains articles on many of the points above.

Software and code

Policy information about availability of computer code

Data collection	Face++ was used to place landmarks on face photographs. Procrustes superimposition was performed using MorphoJ and pairwise inter-landmark distance (ILD) calculation was carried out using R v.4.0.4.
Data analysis	PLINK v1.9 was used to run genome-wide association study. SHAPEIT2 was used to phase chip genotypes data of all samples, and IMPUTE2 was used to impute variants. FUMA was used to annotate SNPs.

For manuscripts utilizing custom algorithms or software that are central to the research but not yet described in published literature, software must be made available to editors and reviewers. We strongly encourage code deposition in a community repository (e.g. GitHub). See the Nature Research guidelines for submitting code & software for further information.

Data

Policy information about availability of data

All manuscripts must include a data availability statement. This statement should provide the following information, where applicable:

- Accession codes, unique identifiers, or web links for publicly available datasets
- A list of figures that have associated raw data
- A description of any restrictions on data availability

Raw genotype or phenotype data cannot be made available due to restrictions imposed by the ethics approval. Summary statistics obtained here have been deposited at GWAS central and will be available at the URL http://www.gwascentral.org/study/HGVST5029 during the Autumn 2022 release.

Field-specific reporting

Please select the one below that is the best fit for your research. If you are not sure, read the appropriate sections before making your selection.

☒ Life sciences ☐ Behavioural & social sciences ☐ Ecological, evolutionary & environmental sciences

For a reference copy of the document with all sections, see nature.com/documents/nr-reporting-summary-flat.pdf

Life sciences study design

All studies must disclose on these points even when the disclosure is negative.

Sample size	6,486 (Colombia, N=1,407; Brazil, N=674; Chile, N=2,003; Mexico, N=1,203 and Peru, N=1,199) individuals from the Consortium for the Analysis of the Diversity and Evolution of Latin America (CANDELA consortium) were included in frontal photographs collection.
Data exclusions	Face images with attribute values indicative of poor quality (e.g. blurriness, head pose) were excluded.
Replication	We examined replication in two independent data samples: one Chinese and one European. We were able to replicate 26 of the 33 novel regions in either replication sample.
Randomization	Randomization of participants to experimental groups was not applicable for our study, as all the participants in the cohort were analyzed in the same way.
Blinding	Investigators were blinded to group allocation during data collection and analysis

Reporting for specific materials, systems and methods

We require information from authors about some types of materials, experimental systems and methods used in many studies. Here, indicate whether each material, system or method listed is relevant to your study. If you are not sure if a list item applies to your research, read the appropriate section before selecting a response.

Materials & experimental systems

n/a	Involved in the study	
☒	☐	Antibodies
☒	☐	Eukaryotic cell lines
☒	☐	Palaeontology and archaeology
☒	☐	Animals and other organisms
☐	☒	Human research participants
☒	☐	Clinical data
☒	☐	Dual use research of concern

Methods

n/a	Involved in the study	
☒	☐	ChIP-seq
☒	☐	Flow cytometry
☒	☐	MRI-based neuroimaging

Human research participants

Policy information about studies involving human research participants

Population characteristics	Adult subjects of both sexes aged between 18 and 45 years old were recruited.
Recruitment	Recruitment took place mainly in five locations: México City (México), Medellín (Colombia), Lima (Perú), Arica (Chile) and Porto Alegre (Brazil). With the exception of Chile, most subjects recruited in these cities were students and staff from the universities participating in this research. In Chile about 2/3 of the subjects recruited were professional soldiers. In Brazil ~10% of samples were collected in smaller towns of the states of Rio Grande do Sul, Bahia and Rondonia.
Ethics oversight	Ethical approval was obtained from the Universidad Nacional Autónoma de México (México), Universidad de Antioquia (Colombia), Universidad Perúana Cayetano Heredia (Perú), Universidad de Tarapacá (Chile), Universidade Federal do Rio Grande do Sul (Brazil) and University College London (UK).

Note that full information on the approval of the study protocol must also be provided in the manuscript.
